# Hyocholic acid species as novel biomarkers for metabolic disorders

**DOI:** 10.1038/s41467-021-21744-w

**Published:** 2021-03-05

**Authors:** Xiaojiao Zheng, Tianlu Chen, Aihua Zhao, Zhangchi Ning, Junliang Kuang, Shouli Wang, Yijun You, Yuqian Bao, Xiaojing Ma, Haoyong Yu, Jian Zhou, Miao Jiang, Mengci Li, Jieyi Wang, Xiaohui Ma, Shuiping Zhou, Yitao Li, Kun Ge, Cynthia Rajani, Guoxiang Xie, Cheng Hu, Yike Guo, Aiping Lu, Weiping Jia, Wei Jia

**Affiliations:** 1 Center for Translational Medicine and Shanghai Key Laboratory of Diabetes Mellitus and Shanghai Key Laboratory of Sleep Disordered Breathing, Shanghai Jiao Tong University Affiliated Sixth People’s Hospital, Shanghai, China; 2 School of Chinese Medicine, Hong Kong Baptist University, Kowloon Tong, Hong Kong China; 3 Institute of Basic Theory for Chinese Medicine, China Academy of Chinese Medical Sciences, Beijing, China; 4 Department of Endocrinology and Metabolism, Shanghai Jiao Tong University Affiliated Sixth People’s Hospital, Shanghai Diabetes Institute, Shanghai, China; 5 Institute of Basic Research in Clinical Medicine, China Academy of Chinese Medical Sciences, Beijing, China; 6 Department of Pharmacology and Toxicology, Tasly Pharmaceutical Co. Ltd, Tianjin, China; 7 University of Hawaii Cancer Center, Honolulu, HI USA

**Keywords:** Endocrine system and metabolic diseases, Endocrinology

## Abstract

Hyocholic acid (HCA) is a major bile acid (BA) species in the BA pool of pigs, a species known for its exceptional resistance to spontaneous development of diabetic phenotypes. HCA and its derivatives are also present in human blood and urine. We investigate whether human HCA profiles can predict the development of metabolic disorders. We find in the first cohort (*n* = 1107) that both obesity and diabetes are associated with lower serum concentrations of HCA species. A separate cohort study (*n* = 91) validates this finding and further reveals that individuals with pre-diabetes are associated with lower levels of HCA species in feces. Serum HCA levels increase in the patients after gastric bypass surgery (*n* = 38) and can predict the remission of diabetes two years after surgery. The results are replicated in two independent, prospective cohorts (*n* = 132 and *n* = 207), where serum HCA species are found to be strong predictors for metabolic disorders in 5 and 10 years, respectively. These findings underscore the association of HCA species with diabetes, and demonstrate the feasibility of using HCA profiles to assess the future risk of developing metabolic abnormalities.

## Abstract (web summary)

The early identification of metabolic disorders could improve or prevent overt disease. Here the authors show that the circulating concentration of hyocholic acid (HCA) species is decreased in the context of obesity and diabetes and increased after gastric bypass surgery in humans, and further that serum HCA species are predictive of metabolic outcomes in healthy individuals.

## Introduction

Metabolism-related disorders, such as obesity, type 2 diabetes mellitus (T2DM), nonalcoholic fatty liver disease (NAFLD), stroke, and cardiovascular disease (CVD), have reached epidemic proportions and thus, become key areas of clinical and translational research over recent decades^1–3^. However, common biomarkers used for the early detection and differential diagnosis of these metabolic diseases have been challenged due to high inter-individual variabilities. For example, not all individuals with obesity develop subsequent metabolic abnormalities, and ~25–40% of them can remain healthy throughout their lives^4–7^. Therefore, the identification of high-risk individuals at an early stage is critical for the prevention and control of metabolic diseases.

Bile acids (BAs) have been linked predominantly to cholesterol metabolism in the liver and the stimulation of cholesterol, fat-soluble vitamins, and lipid absorption from the intestines. Recently, BAs have gained increasing recognition as important signaling molecules that regulate triglycerides (TG), cholesterol, glucose, and energy homeostasis^8–12^. For example, dietary supplements of BA increased energy expenditure and prevented the development of high fat-induced obesity and insulin resistance in mice^13–15^. Plasma chenodeoxycholic acid (CDCA), cholic acid (CA) (primary BAs), and deoxycholic acid (DCA, a secondary BA) concentrations were found to be positively associated with homeostasis model assessment of insulin resistance (HOMA-IR) and negatively associated with glucose infusion rate in healthy volunteers, patients with T2DM, and non-diabetic individuals with obesity^16^. Our group has found that CDCA% (percentage of CDCA relative to total BA) was significantly higher in diabetic individuals with obesity compared to those with normal glucose tolerance and was positively correlated with body mass index (BMI), hemoglobin A1c (HbA1c), TG, and low-density lipoprotein cholesterol (LDL-c), while negatively correlated with high-density lipoprotein cholesterol (HDL-c), and diabetes duration. Obese diabetics with higher baseline CDCA% were more prone to achieve remission 2 years after gastric bypass surgery^17^.

Pigs are unique mammalian species with exceptionally strong resistance to the development of metabolic diseases such as T2DM, NAFLD, and CVD, despite their being raised under diabetes inducing conditions. There is a distinct difference between the BA profiles of pigs and humans that pigs have a large proportion of hyocholic acid (HCA) species (>75%) whereas humans have a much lower percentage (~3%)^18,19^. Due to the important physiological and metabolic roles of BAs, we were motivated to assess the association of circulating HCA species with the development of metabolic disorders in humans.

Here, we show both obesity and diabetes are associated with lower serum and fecal concentrations of HCA species in two clinical cohorts. Serum HCA levels increase in the patients after gastric bypass surgery and could predict the remission of diabetes 2 years after surgery. Two independent prospective cohorts show that serum HCA species are strong predictors for metabolic disorders in 5 or 10 years.

## Results

### Obesity and diabetes were associated with lower serum concentrations of HCA species

To evaluate the association between HCA species and diabetes, we conducted targeted serum BA profiling analyses in a cohort consisting of 1107 participants (610 men and 497 women) selected from the Shanghai Obesity Study (SHOS)^20^. The participants were separated into three groups: healthy lean (HL, *n* = 585), healthy overweight/obese (HO, *n* = 419), and overweight/obese with T2DM (newly diagnosed, drug naive) (OD, *n* = 103). Key clinical metabolic markers were significantly different between any two of the three groups (Supplementary Table 1). The BA measurement was carried out in the laboratory of Shanghai Jiao Tong University Affiliated Sixth People’s Hospital (Lab 1), and a total of 23 BAs met the quality control criteria and were quantified. The three groups had similar total BA levels in all, male and female subjects (Supplementary Fig. 1). The alterations of the different BA species were then compared, the levels of which were the concentration summation of individual unconjugated and conjugated BAs as shown in Supplementary Table 2. The term “HCA species” in this case, indicated HCA, hyodeoxycholic acid (HDCA), glycohyocholic acid (GHCA), and glycohyodeoxycholic acid (GHDCA). The results showed that the HCA species and DCA species were significantly decreased in HO and OD relative to HL. Pairwise Spearman correlation analysis (Supplementary Fig. 2) showed that HCA species inversely correlated with BMI, fasting and post-load glucose, insulin levels and insulin resistance shown by HOMA-IR. DCA species were also correlated with BMI, post-load glucose and insulin levels. These results implied that the levels of HCA species and DCA species were related to glucose metabolism and regulation.

**Fig. 1:**
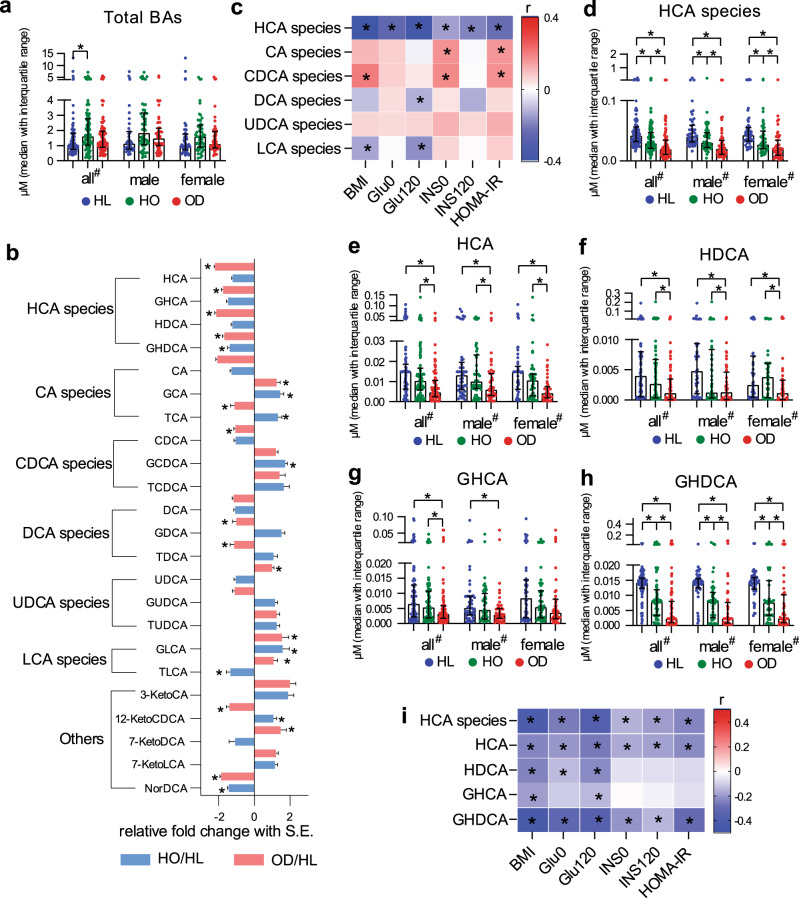
Performances of HCA species and other BA species in the first cross-sectional study. The data were based on a matched cohort, including healthy lean (HL, *n* = 103), healthy overweight/obese (HO, *n* = 103) and overweight/obese with T2DM (OD, *n* = 103) groups. **a** Total BAs based on all (male and female, *n* = 309), male (*n* = 153) and female (*n* = 156) samples. **b** Fold changes of 23 BAs in HO and OD groups relative to the mean values of HL group. **c** Heatmaps of Spearman correlation coefficients of 6 BA species with representative metabolic markers. **d**–**h** Group differences of total and individual HCA species. **i** Heatmaps of Spearman correlation coefficients of total and individual HCA species with representative metabolic markers. Data are expressed as median with interquartile range (**a**, **d**–**h**), and mean with SE (**b**). In the bar plots (**a**, **b**, **d**–**h**), * and # indicate the statistical significance (*p* < 0.05) between two groups and among three groups, respectively, based on two-sided Kruskal–Wallis test. In the heatmaps (**c**, **i**), *r* value indicates Spearman correlation coefficient, and * indicates the statistical significance (*p* < 0.05) based on Spearman correlation.

From HL to HO to OD, the participants had increasingly older age, higher BMI, and a lower ratio of male/female (although the sex ratios were not significantly different among groups) (Supplementary Table 1). To eliminate the confounding effects of age, sex, and BMI, we selected 103 older participants with higher BMI, and more women from the HL and HO groups to better match the 103 participants in the OD group. After this selection, all three groups had matched age and sex ratios, and HO and OD also had matched BMI (Table 1). These matched samples showed higher levels of total BAs in the HO group compared to the HL group (Fig. 1a). Among BA species, HCA species remained at low levels in HO and OD relative to HL (Supplementary Table 3, Fig. 1b), while the levels of DCA species showed no significant difference between groups. Although CDCA species and lithocholic acid (LCA) species showed consistent changes in HO and OD relative to HL, the individuals of each species changed inconsistently (Fig. 1b). Thus, HCA species were distinctive among these BA species. Consistent with the observations in all samples from this cohort, the levels of HCA species still showed an inverse correlation with clinical markers in these matched samples, while levels of DCA species did not (Fig. 1c). These results highlighted the importance of HCA species among BA species in obesity and diabetes.

**Table 1:** Metabolic markers of matched healthy lean (HL), healthy overweight/obese (HO) and overweight/obese with T2DM (OD) subjects in the first cross-sectional study.

	Healthy lean (HL) (*n* = 103)	Healthy overweight/obese (HO) (*n* = 103)	Overweight/obese with T2DM (OD) (*n* = 103)
*n* (M/F)	103 (49/54)	103 (52/51)	103 (52/51)
Age (year)	54 ± 6	53 ± 10	53 ± 10
BMI (kg/m^2^)	21.62 ± 1.77	28.71 ± 4.42*	29.02 ± 3.46*
Glu0 (mmol/L)	5.3 ± 0.5	5.3 ± 0.6	8.2 ± 2.5*^#^
Glu120 (mmol/L)	5.6 ± 1.2	6.1 ± 1.4*	14.4 ± 4.0*^#^
Ins0 (mU/L)	5.21 ± 2.45	11.61 ± 11.94*	16.40 ± 19.36*^#^
Ins120 (mU/L)	28.12 ± 18.50	52.93 ± 55.52*	76.42 ± 57.44*^#^
HbA1c (%)	5.4 ± 0.4	5.4 ± 0.5	7.1 ± 1.3*^#^
HOMA-IR	1.17 ± 0.54	2.77 ± 3.31*	6.03 ± 7.81*^#^
TC (mmol/L)	5.18 ± 1.08	4.85 ± 1.13*	6.21 ± 1.67*^#^
TG (mmol/L)	1.31 ± 0.6	1.34 ± 0.63	2.54 ± 2.39*^#^
HDL-c (mmol/L)	1.67 ± 0.34	1.36 ± 0.25*	1.19 ± 0.24*^#^
LDL-c (mmol/L)	2.57 ± 0.51	2.83 ± 0.43*	3.19 ± 0.84*^#^
SP (mmHg)	116 ± 10	119 ± 15	138 ± 20*^#^
DP (mmHg)	72 ± 7	75 ± 11*	83 ± 13*^#^
HeartRate (bpm)	74 ± 7	75 ± 7	78 ± 6*^#^
ALT (U/L)	23.5 ± 12.5	25.4 ± 12.4	28.9 ± 19.6
AST (U/L)	21.1 ± 5.2	21.8 ± 4.6	23.4 ± 11.5

Values were presented as number or mean ± SD. Chi-Square was used to compare sex ratios between groups.

*Glu0* fasting plasma glucose, *Glu120* 2 h plasma glucose, *Ins0* fasting insulin, *Ins120* 2 h insulin, *ALT* alanine aminotransferase, *AST* aspartate aminotransferase, HOMA-IR = Glu0 × Ins0/22.5.

*Two-sided Kruskal–Wallis test *p* < 0.05 when compared with HL.

^#^Kruskal–Wallis test *p* <  0.05 when compared with HO.

Consistent with levels of total HCA species, the levels of individual HCA species (HCA, HDCA, GHCA, and GHDCA) were highest in HL and lowest in OD (Fig. 1d–h of matched samples, and Supplementary Fig. 3 of all samples), with the fold changes of HO/HL and OD/HL for total HCA species (0.76 and 0.52, respectively), HCA (0.82 and 0.45), HDCA (0.81 and 0.46), GHCA (0.68 and 0.57), and GHDCA (0.73 and 0.60) were determined in the matched samples. Individual HCA species remained inversely correlated with clinical markers across all and matched samples (Supplementary Fig. 4 and Fig. 1i). The results suggested that metabolic disorders including obesity and diabetes were associated with lower concentrations of HCA species in serum.

### Pre-diabetes and diabetes were associated with lower concentrations of HCA species in serum and feces

**Fig. 2:**
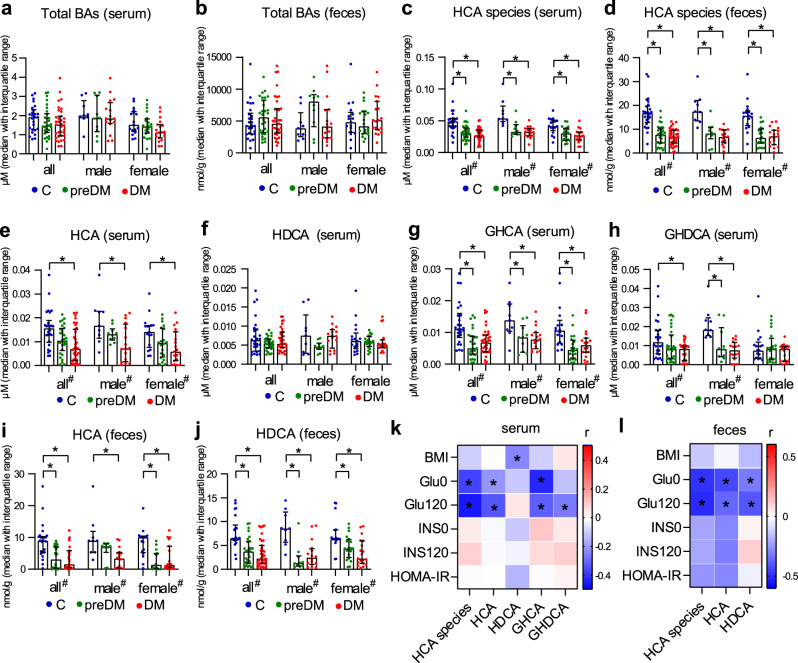
Performance of HCA species in the second cross-sectional study. **a**–**d** Total BAs and total HCA species in serum and feces of healthy control (C, *n* = 26), pre-diabetes (preDM, *n* = 30) and diabetes (DM, *n* = 35) groups. **e**–**j** Individual HCA species in the three groups in all (*n* = 91), male (*n* = 35) and female (*n* = 56) samples. **k**, **l** Heatmaps of Spearman correlation coefficients of total and individual HCA species in serum and feces with representative metabolic markers. Data are expressed as median with interquartile range (**a**–**j**). In the bar plots (**a**–**j**), * and # indicate the statistical significance (*p* < 0.05) between two groups and among three groups, respectively, based on two-sided Kruskal–Wallis test. In the heatmaps (**k**, **l**), *r* value indicates Spearman correlation coefficient, and * indicates the statistical significance (*p* < 0.05) based on Spearman correlation. HCA species in serum is the concentration summation of HCA, HDCA, GHCA, and GHDCA. HCA species in feces is the concentration summation of HCA and HDCA.

We then confirmed the findings above in a separate cohort that focused on diabetes development. The second cohort consisted of 91 participants (35 men and 56 women) including 26 healthy, 30 pre-diabetic and 35 newly diagnosed diabetic individuals (drug naive). In addition to serum samples, fecal samples were collected to evaluate the association between HCA species and metabolic disorder. The clinical markers showed that the HbA1c and fasting and post-load blood glucose levels of patients with pre-diabetes and diabetes were significantly higher than those of healthy controls (Supplementary Table 4). The BA results showed that no significant group differences were found in serum and fecal total BAs (Fig. 2a, b). Compared with healthy controls, the patients with pre-diabetes and diabetes had lower levels of total HCA species in both serum and feces for all, male and female subjects (Fig. 2c, d). The group differences were greater in feces than in serum. As expected, individual HCA species showed similar group differences (Fig. 2e–j, and Supplementary Tables 5, 6). The concentrations of fecal GHCA and GHDCA are not shown as they were below the detection limit. Total and individual HCA species in serum and feces had strong inverse correlations with fasting and post-load blood glucose levels (Fig. 2k, l).

### Gastric bypass surgery increased serum concentrations of HCA species in the patients with metabolic disorders

**Fig. 3:**
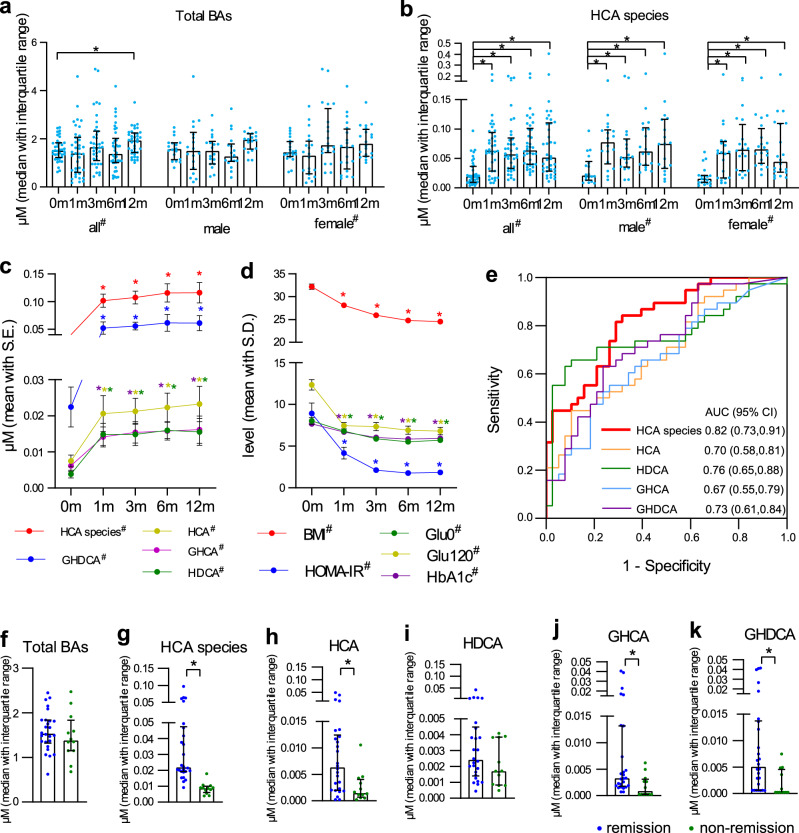
Performances of surgery-induced changes of HCA species in the gastric bypass surgery intervention study. **a**, **b** Total BAs and total HCA species serum concentrations before and after gastric bypass surgery in all (*n* = 38), male (*n* = 18) and female (*n* = 20) patients with obesity and diabetes. **c** Serum concentrations of total and individual HCA species before and after surgery. **d** BMI and glycemic markers before and after surgery. **e** ROC analysis of the changes (12 months vs. baseline) of total and individual HCA species. **f**–**k** The serum concentrations of total BAs, total HCA species, and individual HCA species at baseline (0 month) in the remission group (*n* = 26) and the non-remission group (*n* = 12). Data are expressed as median with interquartile range (**a**, **b**, **f**–**k**), mean with SE (**c**) and mean with SD (**d**). * indicates statistical significance (*p* < 0.05) based on (**a**–**d**) two-sided Wilcoxon signed-rank test when compared with baseline (0 month), and (**f**–**k**) two-sided Mann–Whitney *U* test when comparing remission and non-remission groups.

We further studied the changes of HCA species in patients with obesity and diabetes after Roux-en-Y gastric bypass (RYGB) surgery. Thirty-eight patients who received RYGB were examined before and at 1, 3, 6, and 12 months post-surgery (Table 2). Serum concentration of total BAs gradually increased after RYGB surgery, and became significantly higher than baseline at 12 months post-operation (Fig. 3a). The concentrations of total and individual HCA species in the serum increased drastically 1 month after the surgery (FC = 2.66, 2.75, 3.85, 2.27, and 2.30 for total HCA species, HCA, HDCA, GHCA, and GHDCA, respectively) and maintained minor increases afterward (Fig. 3b, c and Supplementary Table 7). Improvements in BMI, fasting and post-load blood glucose levels, HbA1c, and decreased insulin resistance occurred throughout the post-surgical 12 months (Fig. 3d). The receiver operating characteristic (ROC) analysis showed that the area under the curves (AUCs) for the 12-month changes of total HCA species, HCA, HDCA, GHCA, and GHDCA were 0.82, 0.70, 0.76, 0.67, and 0.73, respectively (Fig. 3e), providing evidence that HCA levels had potential prediction capability for the metabolic outcome of RYGB surgery.

**Table 2:** Metabolic markers of patients with diabetes at baseline (0 m) and 1, 3, 6, and 12 months after surgery in the gastric bypass surgery intervention study.

	Baseline (0 month) (*n* = 38)	1 month after (*n* = 38)	3 months after (*n* = 38)	6 months after (*n* = 38)	12 months after (*n* = 38)
*n* (M/F)	38 (18/20)	38 (18/20)	38 (18/20)	38 (18/20)	38 (18/20)
Age (year)	45 ± 13	–	–	–	50 ± 13
BMI (kg/m^2^)	32.21 ± 3.84	28.10 ± 3.53*	25.95 ± 3.21*	24.78 ± 2.91*	24.53 ± 2.71*
Waist (cm)	107.18 ± 12.52	95.89 ± 9.80*	89.89 ± 9.78*	86.63 ± 9.08*	86.08 ± 8.49*
Glu0 (mmol/L)	8.0 ± 2.5	6.8 ± 1.8*	5.9 ± 1.6*	5.5 ± 1.1*	5.7 ± 1.1*
Glu120 (mmol/L)	12.3 ± 4.0	7.5 ± 2.5*	7.4 ± 3.1*	6.9 ± 3.0*	6.8 ± 2.6*
Ins0 (mU/L)	25.49 ± 22.38	13.03 ± 10.25*	8.02 ± 5.00*	7.20 ± 4.29*	7.28 ± 4.18*
Ins120 (mU/L)	105.24 ± 79.12	20.91 ± 17.38*	23.81 ± 20.74*	31.77 ± 32.02*	24.17 ± 19.51*
TC (mmol/L)	7.7 ± 1.9	6.7 ± 1.2*	6.0 ± 1.2*	5.9 ± 0.6*	5.9 ± 1.2*
TG (mmol/L)	2.6 ± 3.1	1.5 ± 0.6*	1.2 ± 0.6*	1.1 ± 0.6*	1.0 ± 0.6*
HDL-c (mmol/L)	1.02 ± 0.16	0.96 ± 0.19	1.03 ± 0.19	1.16 ± 0.19*	1.26 ± 0.27*
LDL-c (mmol/L)	3.10 ± 0.91	3.15 ± 0.90	2.61 ± 0.61*	2.49 ± 0.74*	2.49 ± 0.63*
HbA1c (%)	7.7 ± 1.7	6.7 ± 1.1*	6.0 ± 1.0*	5.8 ± 0.8*	5.9 ± 1.0*
HOMA-IR	8.92 ± 7.65	4.16 ± 4.36*	2.13 ± 1.52*	1.79 ± 1.20*	1.86 ± 1.16*

Values were presented as number or mean ± SD.

*Two-sided Wilcoxon paired samples signed-rank *p* < 0.05 when compared with baseline (0 month).

Two years after surgery, a total of 26 individuals remained in remission, while 12 had diabetes recurrence. The remission group had lower HbA1c levels and fasting blood glucose levels at baseline compared to the non-remission group (Supplementary Table 8). Regarding serum BAs (Supplementary Table 9), total BAs were comparable between both groups (Fig. 3f). The levels of total HCA species and most individual BAs, HCA, GHCA, and GHDCA, were significantly higher in the remission group compared to the non-remission group (Fig. 3g–k). The level of HDCA was also relatively higher in the remission group (Fig. 3i), although such difference did not reach statistical significance due to intra-group variations. The binary logistic regression models indicated that higher levels of total HCA species before surgery were associated with a higher probability of maintaining diabetes remission 2 years after RYGB. The odds ratio was 0.74 (95% CI, 0.58–0.95) after adjustment for age, gender, HbA1c level, and fasting blood glucose level. However, some individuals achieved remission at 6 or 12 months but relapsed at 2 years. We therefore divided the individuals into four groups based on their clinical indices of remission or non-remission at 6, 12, and 24 months post-surgery. The four groups include (1) remission at 6 months post-surgery without recurrence of diabetes; (2) remission at 12 months without recurrence of diabetes; (3) remission at 6 or 12 months but relapsed at 12 or 24 months; and (4) non-remission after surgery. The results (Supplementary Fig. 5) showed that the initial remitters who relapsed at 12 or 24 months had relatively low levels of HCA species pre-surgery, similar to the non-remitters. Differently, the individuals in the group (2) had relatively high levels of HCA species at baseline, similar to the remitters. Thus, pre-surgery levels of HCA species may be useful in predicting long-term remission after surgery instead of short-term remission.

### Serum HCA species were strong predictors for future metabolic outcomes in healthy individuals

**Fig. 4:**
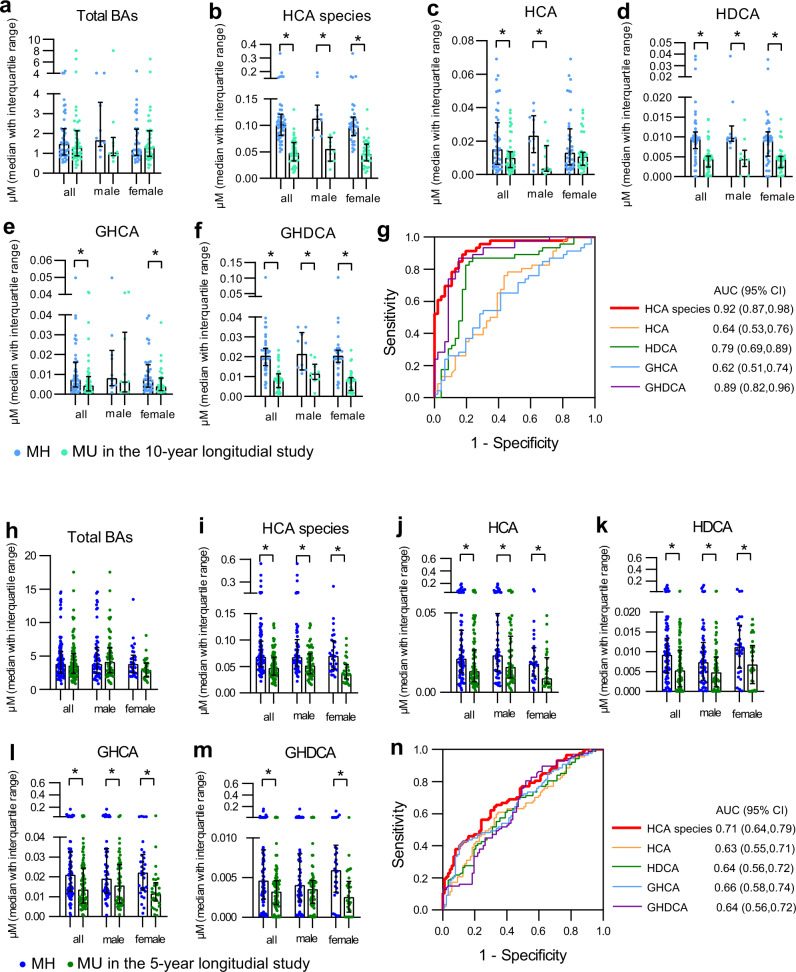
Performances of HCA species in the 10-year and 5-year longitudinal studies. In the 10-year longitudinal study, **a**–**f** total BAs, total and individual HCA species in the serum of matched all (*n* = 92), male (*n* = 20), and female (*n* = 72) individuals in future metabolically healthy (MH) (*n* = 46) and metabolically unhealthy (MU) (*n* = 46) groups. **g** ROC analyses of total and individual HCA species for the metabolic health longitudinal study. HCA species is the concentration summation of HCA, HDCA, GHCA, and GHDCA. In the 5-year longitudinal study, **h**–**m** total BAs, total and individual HCA species in the serum of matched all (*n* = 174), male (*n* = 114), and female (*n* = 60) individuals in future MH (*n* = 87) and MU (*n* = 87) groups. **n** ROC analyses of total and individual HCA species for the metabolic health longitudinal study. HCA species is the concentration summation of HCA, HDCA, GHCA, GHDCA, THCA, and THDCA. Data are expressed as median with interquartile range (**a**–**f**, **h**–**m**). *indicates statistical significance (*p* < 0.05) based on two-sided Mann–Whitney *U* test when comparing MH and MU.

To evaluate the association between HCA species and future metabolic health, we selected 132 subjects (36 men and 96 women) from the Shanghai Diabetes Study^21^. All of them were metabolically healthy (MH, defined in the “Methods” section) at their enrollment. After 10 years, 86 participants became metabolically unhealthy (MU, defined in the “Methods” section), and 46 remained MH. At baseline, the future MU group were older, had higher BMI and more men than the future MH group (although group differences of sex ratio did not reach statistical significance), however, the major metabolic markers were similar between the two groups (Supplementary Table 10). To eliminate the confounding effects of age, sex and BMI, we chose 46 younger participants with lower BMI and comprised of more women, from the MU group to match the 46 participants in the MH group (Supplementary Table 11). When samples from all participants were considered, the concentrations of total BAs in serum were comparable between the MH and MU groups, but the concentrations of total and individual HCA species were significantly lower in the MU than the MH group (Supplementary Fig. 6 and Supplementary Table 12). Age-, sex-, and BMI-matched samples yielded similar results as all group samples did (Fig. 4a–f, Supplementary Table 13), suggesting that the baseline differences in HCA species between MH and MU groups were independent of age, sex, and BMI. Binary logistic regression analysis of all group samples showed that the association between HCA species and future MU outcome were (odds ratio (95% CI)) 0.89 (0.86, 0.93), 0.91 (0.87, 0.94), 0.90 (0.84, 0.96), 0.92 (0.85, 0.99), 0.52 (0.40, 0.69) and 0.90 (0.86, 0.94) for total HCA species, HCA, GHCA, HDCA, and GHDCA, respectively (*p* < 0.05 for all, adjusted for age, sex, and BMI) (Supplementary Fig. 7). The ROC curve analysis showed that the total HCA species (red line in Fig. 4g) had the highest AUC of 0.92, and the AUCs of individual HCA species ranged from 0.62 to 0.89, providing supporting evidence for using total and individual HCA species as predictors for future metabolic outcome.

### Validation study of HCA species as predictive biomarkers for future metabolic outcome

To validate the predictive capability of serum HCA species for future metabolic outcome, we further collected serum samples from an independent cohort from Beijing, China. BA profiling was performed in an independent laboratory in Shenzhen, China (Lab 2), and the assessment instruments and methods were slightly different from those applied in the previous four cohorts in the Lab 1. A total of 207 subjects (117 men and 90 women) were selected. These participants were metabolically healthy in the year of 2011, and after 5 years, 90 of them (60 men and 30 women) became MU, while 117 (57 men and 60 women) remained metabolic healthy. The major metabolic markers were similar between the two groups in 2011 (Supplementary Table 14). We further selected 87 samples (57 men and 30 women) from MH and MU groups, respectively, with matched age and metabolic markers as a matched cohort (Supplementary Table 15).

A total of 27 BAs were quantified in Lab 2, among which, 20 were the same BAs that were also detected in Lab 1, and 3 BAs including 3-ketoCA, 7-ketoLCA, and 12-ketoCDCA were missed for lack of chemical standards. Notably, THCA and THDCA were quantified in Lab 2 due to the high sensitivity of the mass spectrometry, while these two metabolites did not pass the quality control in Lab 1 (>40% lower than limit of quantification). Thus, the HCA species in this cohort were defined as the concentration summation of HCA, HDCA, GHCA, GHDCA, THCA, and THDCA.

The samples from all participants (Supplementary Table 16 and Supplementary Fig. 8) and the matched cohort (Supplementary Table 17 and Fig. 4h–m) showed similar results, that is, the concentration of total BAs was comparable between the MH and MU groups. The concentrations of total HCA species and four individual BAs including HCA, HDCA, GHCA, and GHDCA were significantly lower in the MU than the MH group. The levels of THCA and THDCA were also lower in the MU group compared to the MH group, although without significant difference (Supplementary Fig. 9). The ROC analysis showed that the total HCA species (the summation of HCA, HDCA, GHCA, GHDCA, THCA, and THDCA) (red line in Fig. 4n) had the highest AUC of 0.71, and the AUCs of the four individual HCA species ranged from 0.63 to 0.66. We further carried out the ROC analysis in the summation of HCA, HDCA, GHCA, and GHDCA, and the AUC was 0.72, higher than that of total HCA species of six BAs (Supplementary Fig. 10). These results validated our previous results that total and individual HCA species had robust predictive capability for future metabolic outcome. It also suggested that the concentrations of the four individual HCA species, HCA, HDCA, GHCA, and GHDCA, as well as their summation were good predictors in humans, while THCA and THDCA were not.

## Discussion

BAs play a crucial role in dietary lipid digestion and absorption, and also act as signaling molecules to regulate glucose and lipid metabolism through activation of several receptors including the nuclear hormone receptor farnesoid X receptor (FXR) and the G-protein-coupled receptor, TGR5. In recent years, BA derivatives have been extensively evaluated as potential therapeutic agents for metabolic diseases^22,23^. Consistent with published studies^24^, our data showed that total BA concentrations were higher in obese patients and positively correlated with BMI. We also observed that serum total BAs were decreased with increases in blood glucose levels, and the ratio of 12α-hydroxy- to non-12α-hydroxy-BA was inversely associated with insulin sensitivity in T2DM patients^25–29^. Activation of the BA alternative synthetic pathway leading to increased production of non-12α-hydroxy-BA resulted in beneficial effects on glucose and lipid metabolism^15,28,30–32^. It is worth noting, however, the levels of total BAs and BA pool composition in T2DM patients differed among studies. Some other studies have shown that there was no change in fasting total BAs in T2DM compared with non-diabetic controls, while postprandial total and glycol-BAs increased in T2DM^25^. The differences in BA results among studies may be due to the differences in methodological parameters (e.g., feeding state and time of T2DM onset)^16^. Thus, it is necessary to select specific BA species, such as HCA species, which are closely associated with glycemic control for comparisons between patient groups.

A recent 5-year prospective study in patients with pre-diabetes reported similar results regarding the association between low HCA levels and the risk of new-onset diabetes^33^. We found in this study that lower serum concentrations of HCA species were associated with diabetes and were closely related to glycemic markers. We aimed to evaluate the clinical performance of HCA species in not only T2DM patients but also those with obesity and metabolic syndrome. In this study, we enlarged the sample size in several independent cohorts from different cities in China.

In the 5-year longitudinal cohort, THCA and THDCA were detected at baseline but were below detection limits in future MU samples. Meanwhile, these two BAs were not as significantly decreased as the four HCA species. However, THCA and THDCA showed similar stimulation effects on GLP-1 secretion as the other four HCA species in our studies (data were not shown). Thus, taurine conjugated HCA species might be good candidates for pharmacological research, while they could not be clinical biomarkers due to their low serum concentrations.

T2DM is inherently associated with obesity and aging^24^, so we tried to eliminate the confounding effects of BMI and age when evaluating the role of HCA species in T2DM. By matching age and/or BMI between the groups in comparison, we demonstrated that HCA species had direct correlations with glycemic markers and future metabolic outcomes. These results provided evidence that HCA species play critical roles in regulating glucose homeostasis and are protective against the development of T2DM in humans.

We also showed that, compared with healthy controls, patients with pre-diabetes and diabetes had only ~27% lower serum levels of HCA species, but strikingly ~57% lower HCA species in feces, although these patients had similar levels of total BAs in feces as controls. Notably, the patients with pre-diabetes and diabetes had higher BMIs than the healthy controls, which suggest that they may also have altered gut microbiota^34^ as previously discussed.

RYGB surgery is considered a rapid resolution of metabolic disorders. Total and individual HCA species were found significantly increased after RYGB^19^; and among all BAs, the increases in HCA species were the most pronounced and consistent. Our results further highlight the potential predictive value of HCA species for long-term post-operative metabolic outcomes irrespective of the initial remission. Future follow-up studies with large sample sizes and long duration (3 years or more) are necessary to provide more convincing evidence to validate the prognostic value of blood HCA levels for RYGB surgery.

In conclusion, obesity and diabetes were associated with significantly lower levels of HCA species in serum. There were several limitations in the present study. First, the sample size of metabolic surgery study was relatively small. The good performance of HCA species in evaluation and prediction of the surgery outcomes warrant further investigations with larger sample sizes in a multi-center setting. Second, this is a discovery study establishing an associative relationship between HCA species and T2DM. Further studies are needed to investigate the underlying mechanisms.

## Methods

### Human studies

The five human studies reported in this paper were all approved by the Ethics Committee of Shanghai Jiao Tong University Affiliated Sixth People’s Hospital and Institute of Basic Theory of Chinese Medicine, China Academy of Chinese Medical Sciences in accordance with the World Medical Association’s Declaration of Helsinki. Written informed consent was obtained from all participants before recruitment. Clinical parameters were determined at the Shanghai Jiao Tong University Affiliated Sixth People’s Hospital and Institute of Basic Theory of Chinese Medicine, China Academy of Chinese Medical Sciences.

#### Human study 1: cross-sectional study 1

This is a nested case-control study, performed within the SHOS^20^, which was designed to investigate the occurrence and development of the metabolic syndrome and its related diseases. Beginning in 2009, the SHOS recruited participants from four communities in Shanghai, China. The participants that were selected satisfied the criteria for overweight/obese and diabetes. The exclusion criteria were: type 1 diabetes, pregnancy, severe diabetic complications (diabetic retinopathy, diabetic neuropathy, diabetic nephropathy, and diabetic foot); severe hepatic diseases including chronic persistent hepatitis, liver cirrhosis or the co-occurrence of positive hepatitis and abnormal hepatic transaminase; severe organic disease, including cancer, coronary heart disease, renal disease, thyroid disease, myocardial infarction, or cerebral apoplexy; infectious disease; alcoholism; and continuous medication (including weight loss or psychotropic medication) for over 3 days prior to enrollment. According to these criteria, a total of 1107 subjects with fasting serum samples were selected prior to BA analysis. This cohort included 585 HL (329 men and 256 women), 419 HO (229 men and 190 women) and 103 OD (52 men and 51 women) participants (all samples from cohort 1). We further selected 103 subjects from each group with matched age, sex, and BMI as a matched cohort (matched samples from cohort 1).

#### Human study 2: cross-sectional study 2

A group of 91 subjects including 26 healthy controls (9 men and 17 women), 30 individuals with pre-diabetes (10 men and 20 women) and 35 patients with diabetes (16 men and 19 women) were recruited for this study. The exclusion criteria were the same as in human study 1. Fasting sera and fecal samples of 91 participants were collected and stored for later analysis.

#### Human study 3: Gastric bypass surgery intervention study

A total of 38 patients with obesity and diabetes who received RYGB surgery were enrolled in the study^17^. Any patient with a history of open abdominal surgery, a serious disease (such as heart or lung insufficiency) that was incompatible with surgery, an acute T2DM complication, severe alcohol or drug dependency, a mental disorder, type 1 diabetes, secondary diabetes, an unstable psychiatric illness, or who was at a relatively high surgical risk (such as a patient with an active ulcer) was excluded. The fasting serum specimens of these subjects were collected and stored for future analysis before (baseline) and 1, 3, 6, and 12 months after the surgery.

#### Human study 4: a 10-year longitudinal study

A group of 132 subjects (36 men and 96 women) were selected from the Shanghai Diabetes Study, which was intended to assess the prevalence of diabetes and diabetes-associated metabolic disorders in urban Shanghai^21^. All 132 subjects were metabolically healthy at baseline (the year 2000–2001). Ten years later (year 2010–2011), 86 participants (26 men and 60 women) became MU (future MU), and 46 participants (10 men and 36 women) remained healthy (future metabolically healthy). Fasting serum samples of the 132 participants at baseline were collected and stored for future analysis.

#### Human study 5: a 5-year longitudinal study

A group of 207 subjects (117 men and 90 women) were selected from the physical examination centers in Beijing. All 207 subjects were metabolically healthy at baseline (the year 2011). Five years later (the year 2016), 90 participants (60 men and 30 women) became MU (future MU) and 117 participants (57 men and 60 women) remained healthy (future metabolically healthy). Fasting serum samples of the 207 participants at baseline were collected and stored for future analysis.

#### Criteria for lean, overweight/obesity, pre-diabetes, diabetes, metabolically healthy, and unhealthy status

Individuals with BMI < 25 kg/m^2^ were considered lean and those with BMI ≥ 25 were classified as overweight/obese. Individuals with 6.1 mmol/L ≤ fasting blood glucose < 7.0 mmol/L or 7.8 mmol/L ≤ oral glucose tolerance test (OGTT) (2 h) < 11.1 mmol/L were classified as pre-diabetic. Subjects with fasting blood glucose ≥ 7.0 mmol/L and/or OGTT (2 h) ≥ 11.1 mmol/L were classified as diabetic. Subjects were considered “metabolically healthy” if they met all of the following criteria: fasting blood glucose < 6.1 mmol/L, OGTT (2 h) < 7.8 mmol/L and no previous history of diabetes; systolic blood pressure (SP)/diastolic blood pressure (DP) < 140/90 mmHg and no previous history of high blood pressure; fasting plasma TG < 1.7 mmol/L and fasting plasma high-density lipoprotein-cholesterol (HDL-c) ≥ 0.9 mmol/L (men) or ≥1.0 mmol/L (women), and no previous history of high cholesterol (total cholesterol (TC) < 5.18 mmol/L); no history of cardiovascular or endocrine disease^7^. Those who failed to meet all criteria above were classified as “metabolically unhealthy”.

#### Clinical measurements and sample collection

All human samples were collected, stored and measured following the standard operating protocol of the hospital. Participants were given a standard 75-g OGTT after an overnight fast of more than 8 h. Venous blood samples were drawn at 0 (fasting), 30, 60, and 120 min. Fasting and postprandial plasma glucose and insulin levels, fasting serum lipid profiles (TC, TG, HDL-c, LDL-c), blood pressure (SP and DP), waist circumference, BMI, liver, and kidney function tests were determined as previously described^7,35^. The blood samples were centrifuged for plasma or serum collection, and then divided into aliquots and delivered on dry ice to the study laboratory. Fecal samples from the recruited subjects were collected in the sterile feces containers (Cat# 80.9924.014, SARSTEDT). Each sample was either frozen immediately at −80  °C or briefly stored in personal −20 °C freezers before transport to the laboratory within 24 h. All samples were stored in a −80 °C freezer until analysis.

### Quantitative analysis of BAs

BAs were quantified using in-house established methods with minor modifications to improve accuracy (Supplementary Fig. 11, 12, and Supplementary Table 18).

#### Methods of Lab 1

For sample pretreatment, an aliquot of 50 µL serum sample was mixed with 300 µL acetonitrile–methanol (8:2 v/v) containing six internal standards (IS) (D4-GCA, D4-GDCA, D4-CA, D4-UDCA, D4-LCA, and D4-GCDCA, 50 nM for each). The mixture was allowed to stand at 20 °C for 30 min, and was then centrifuged at 13,000 × *g* at 4 °C for 30 min. An aliquot of 300 µL of supernatant was transferred to another tube and then vacuum-dried. A 25 µL volume of acetonitrile–methanol (9/1, v/v) containing 0.01% formic acid was added, and the sample was re-vortexed at 1500 × *g*, 10 °C for 10 min followed by addition of 25 µl water containing 0.01% formic acid. The sample was vortexed again at 1500 × *g*, 10 °C for 10 min, and then centrifuged at 13,000 × *g*, 4 °C for 15 min. The supernatant was used for ultra-performance liquid chromatography–tandem mass spectrometry (UPLC–MS) analysis. For fecal samples, 10 mg of dried samples were accurately weighed and homogenized with 200 µL of a mixture of methanol and water (1:1, v/v) (containing 6 IS: D4-GCA, D4-GDCA, D4-CA, D4-UDCA, D4-LCA, and D4-GCDCA, 50 nM for each) for 5 min. After centrifugation at 13,000 × *g* for 15 min, the supernatant was transferred to a 1.5 mL tube. The residue was reconstituted with 200 µL of a mixture of acetonitrile–methanol (8:2, v/v) (containing 6 IS: D4-GCA, D4-GDCA, D4-CA, D4-UDCA, D4-LCA, and D4-GCDCA, 50 nM for each) for 5 min. After centrifugation at 13,000 × *g* for 15 min, the supernatant was added to the same tube. Each combined supernatant was centrifuged at 13,000 × *g* for 10 min and then, a 60 µL aliquot of the supernatant of each sample was used for UPLC–MS analysis.

For instrumental analysis, an aliquot of standard stock solution was prepared by mixing BA standards for a final concentration of 5 µM each. A series of standard calibration solutions were diluted with desalted serum (depleted of BAs using activated charcoal) for the calibration curve. The calibration curve and the corresponding regression coefficients were obtained by internal standard adjustment. A Waters ACQUITY UPLC system equipped with a binary solvent delivery manager and a sample manager (Waters, Milford, MA) was used throughout the study. The mass spectrometer was a Waters XEVO TQ instrument with an ESI source (Waters Corp., Milford, MA). The entire UPLC−MS system was controlled by MassLynx 4.1 software. All chromatographic separations were performed with an ACQUITY BEH C18 column (1.7 μm, 100 mm × 2.1 mm internal dimensions) (Waters Corp., Milford, MA). The mobile phase consisted of water with 0.01% formic acid (mobile phase A) and acetonitrile/methanol with 0.01% formic acid (9/1, v/v, mobile phase B). The flow rate was 0.45 mL/min with the following mobile phase gradient: 0−0.5 min (5% B), 0.5−1 min (5−20% B), 1−2 min (20−25% B), 2−5.5 min (25% B), 5.5−6 min (25−30% B), 6−8 min (30% B), and 8−9 min (30−35% B), 9−17 min (35−65% B), 17−18 min (65−99% B), 18−19 min (99% B), 19−19.1 min (99−5% B), 19−20 min (5% B). The column was maintained at 45 °C and the injection volume for all samples was 5 μL. The mass spectrometer was operated in negative ion mode with a 2.5 kV capillary voltage. The source and desolvation gas temperature were 150 and 450 °C, respectively. The data were collected with a multiple reaction monitor (MRM), and the cone and collision energy for each BA used the optimized settings from QuanOptimize application manager (Waters Corp., Milford, MA).

#### Methods of Lab 2

For sample pretreatment, a 20 µL serum sample aliquot was mixed with 180 µL acetonitrile–methanol (8:2 v/v) containing 0.2% formic acid and 7 IS (D4-LCA, D4-DCA, D4-GDCA, D4-GCDCA, D4-CA, D4-GCA, D4-TCA, 50 nM for each) in a 96-well plate. It was then mixed at 650 × *g*, 10 °C for 20 min, followed by centrifugation at 13,000 × *g* (4 °C) for 30 min. A 170 µL aliquot of supernatant was transferred to a 96-well plate and then vacuum-dried for 1.5 h. 50 µL of acetonitrile–methanol (8:2 v/v) were added, and the sample was mixed at 650 g, 10 °C for 20 min. 70 µl water were then added. The sample was mixed again at 650 × *g*, 10 °C for 20 min, and then centrifuged at 13,000 × *g*, 4 °C for 30 min. The supernatant was transferred to a new 96-well plate used for UPLC–MS analysis.

For instrumental analysis, an aliquot of standard stock solution was prepared by mixing BA standards for a final concentration of 5 µM each. A series of standard calibration solutions were diluted with 50% methanol for the calibration curve. The calibration curve and the corresponding regression coefficients were obtained using internal standard (D4-LCA, D4-DCA, D4-GDCA, D4-GCDCA, D4-CA, D4-GCA, D4-TCA) adjustment. A Waters ACQUITY UPLC system equipped with a binary solvent delivery manager and a sample manager (Waters, Milford, MA) was used throughout the study. The mass spectrometer was a Waters XEVO TQS instrument with an ESI source (Waters Corp., Milford, MA). The entire LC−MS system was controlled by MassLynx 4.1 software. All chromatographic separations were performed with a CORTECS UPLC C18 column (1.6 μm, 100 mm × 2.1 mm internal dimensions) (Waters Corp., Milford, MA). The mobile phase consisted of water with 5 mM ammonium acetate (mobile phase A) and acetonitrile/methanol (80/20, v/v, mobile phase B). The flow rate was 0.40 mL/min with the following mobile phase gradient: 0−0.5 min (5% B), 0.5−3 min (5−30% B), 3−6 min (30% B), 6−8 min (30–35% B), 8−9 min (35−40% B), 9−10 min (40% B), and 10−15 min (40−75% B), 15−15.5 min (75−100% B), 15.5−16.3 min (100% B), 16.3−16.5 min (100–5% B), 16.5−17 min (5% B). The column was maintained at 30 °C and the injection volume for all samples was 5 μL. The mass spectrometer was operated in negative ion mode with a 2.5 kV capillary voltage. The source and desolvation gas temperature were 150 and 450 °C, respectively. The data were collected with MRM, and the cone and collision energy for each BA used the optimized settings from QuanOptimize application manager (Waters Corp., Milford, MA).

#### Method validation

The two methods for BA quantification were verified independently using commercially available standard human plasma (NIST 1950) and cross-validated between the two labs (Lab 1 and Lab 2). The limit of detection (LOD) and limit of quantification (LOQ) are shown in Supplementary Tables 19, 20. The results (Supplementary Table 21) showed that the intra-batch and inter-batch precision CVs were lower than 20%.

### Statistical analysis

The BA profile raw data acquired using UPLC–MS were processed and quantified using TargetLynx software (Waters Corp., Milford, MA). Manual checking and corrections were carried out in order to ensure data quality. The sample sizes were predetermined by IP4M 2.0 (http://ip4m.cn)^36^. The power was set as 0.85 (significant level 0.05, two tails) and the effect size was set as 0.5. The sample distribution was determined using a Kolmogorov–Smirnov normality test. For statistical comparisons, Mann–Whitney *U* test or Kruskal–Wallis test followed by a pairwise comparison was carried out for comparisons of two or more than two groups, respectively. Wilcoxon signed-rank test was carried out for comparisons of paired sample groups. Spearman’s rank correlation coefficients were calculated to examine the association of BAs and typical clinical measurements. Two-tailed *p* values smaller than 0.05 were considered significant. All the *p* values were corrected for multiple testing by the Benjamini–Hochberg false discovery rate test. ROC analysis was used to test the sensitivity and specificity of total and individual HCA species in the group separation. The ratio of molar sums of BA (Fig. 1b) was the ratio of the level of specific BA for each individual in HO and OD groups to the mean level in HL group. SPSS (V19, IBM, USA), GraphPad Prism (6.0, GraphPad, USA), and IP4M 2.0 were used for statistical analyses and graphic generation.

### Reporting summary

Further information on research design is available in the Nature Research Reporting Summary linked to this article.

## Supplementary information

Supplementary Information

Reporting Summary

## Data Availability

The metabolomics data were deposited and available at Metabolights repository with accession code MTBLS2343. Other data supporting the findings of this study are available from the corresponding authors upon reasonable request. Source data are provided with this paper.

## Source data

Source Data
