# Supplementary material for: Hyocholic acid species as novel biomarkers for metabolic disorders
Source: Nat Commun. 2021 Mar 5;12:1487. doi: 10.1038/s41467-021-21744-w (PMC7935989; doi:10.1038/s41467-021-21744-w)
Supplement: Supplementary file 1 — Supplementary Information [file 41467_2021_21744_MOESM1_ESM.pdf]

Hyocholic acid species as novel biomarkers for metabolic disorders

Supplementary material

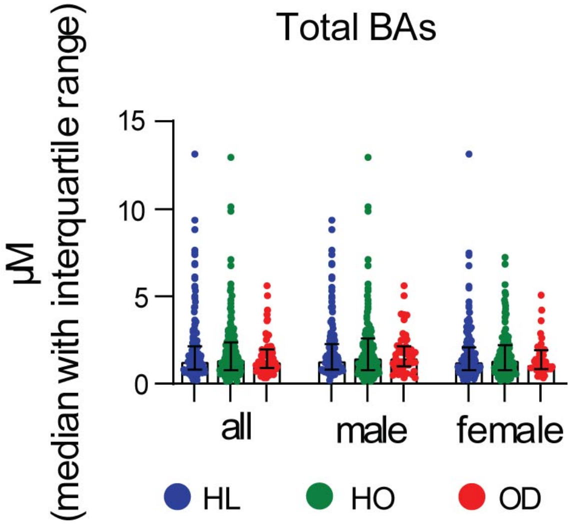

Supplementary Fig. 1. Serum concentrations of total BAs for HL (n=585), HO (n=419), and OD (n=103) groups in the first cross-sectional study. Data are expressed as median with interquartile range.

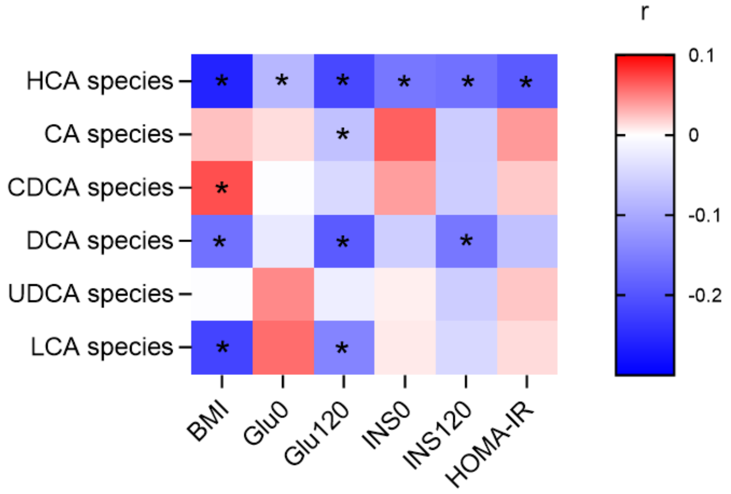

Supplementary Fig. 2. Heatmaps of Spearman correlation coefficients of 6 BA species with representative metabolic markers (all samples from cohort 1). r value indicates Spearman correlation coefficient, and * indicates the statistical significance (p<0.05) based on Spearman correlation.

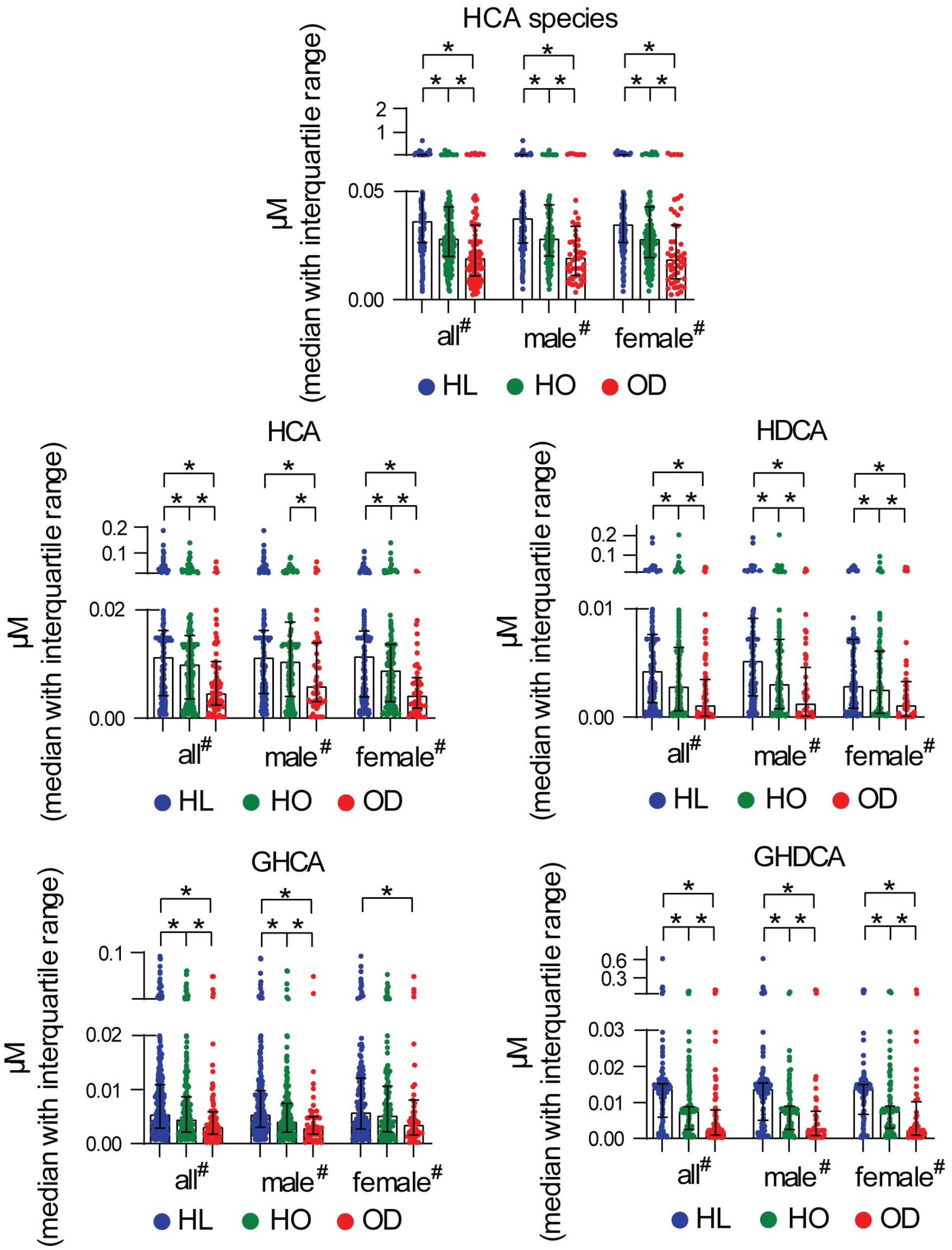

Supplementary Fig. 3. Serum concentrations of total and individual HCA species for HL (n=585), HO (n=419), and OD (n=103) groups in the first cross-sectional study. Data are expressed as median with interquartile range. * and # indicate the statistical significance (*p*<0.05) between 2 groups and among 3 groups, respectively, based on two-sided Kruskal-Wallis test.

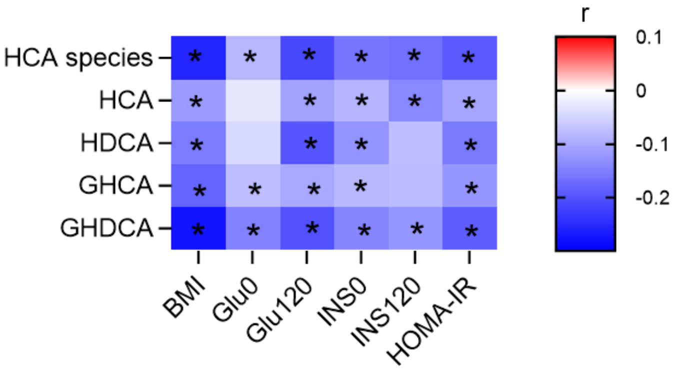

Supplementary Fig. 4. Heatmaps of Spearman correlation coefficients of total and individual HCA species with representative metabolic markers (all samples from cohort 1). r value indicates Spearman correlation coefficient, and * indicates the statistical significance (*p*<0.05) based on Spearman correlation.

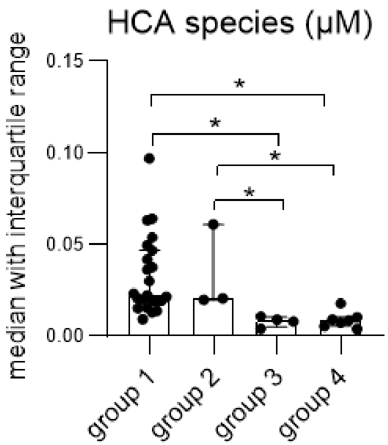

Supplementary Fig. 5. The baseline levels of HCA species of the subjects with different remission situations. Data are expressed as median with interquartile range. * the statistical significance (*p*<0.05) between 2 groups based on two-sided Kruskal-Wallis test.

group 1 (n=23): remission at 6 months post-surgery without recurrence of diabetes;

group 2 (n=3): remission at 12 months without recurrence of diabetes;

group 3 (n=4): remission at 6 or 12 months but relapsed at 12 or 24 months;

group 4 (n=7): non-remission after surgery.

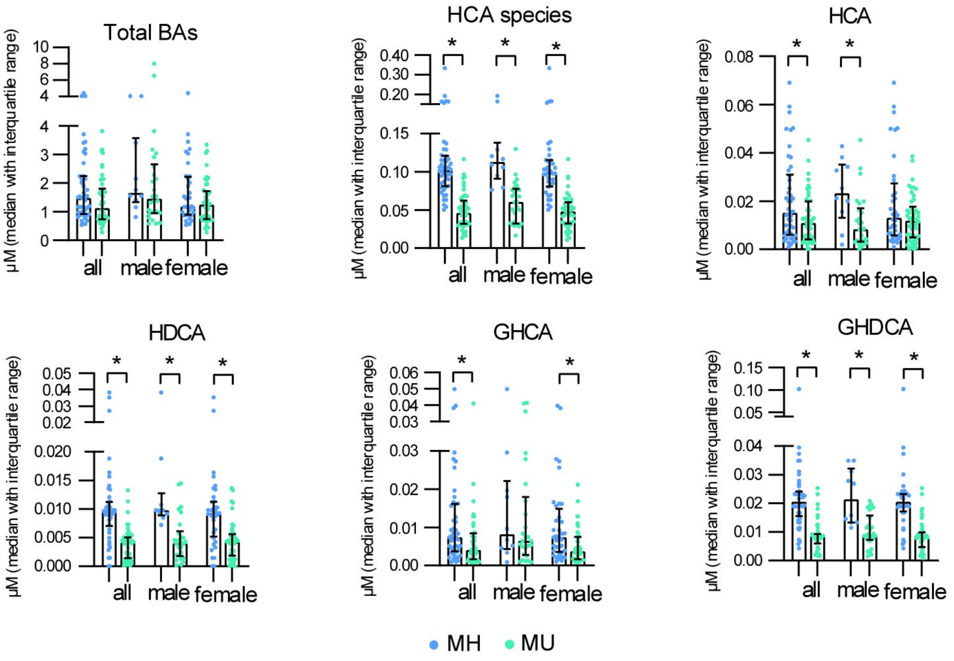

Supplementary Fig. 6. Serum concentrations of total BAs and total and individual HCA species for future metabolically healthy (MH, n=46) and unhealthy (MU, n=86) groups in the 10-year longitudinal study. Data are expressed as median with interquartile range. * indicate *p*<0.05 based on two-sided Mann-Whitney U test comparing MH and MU.

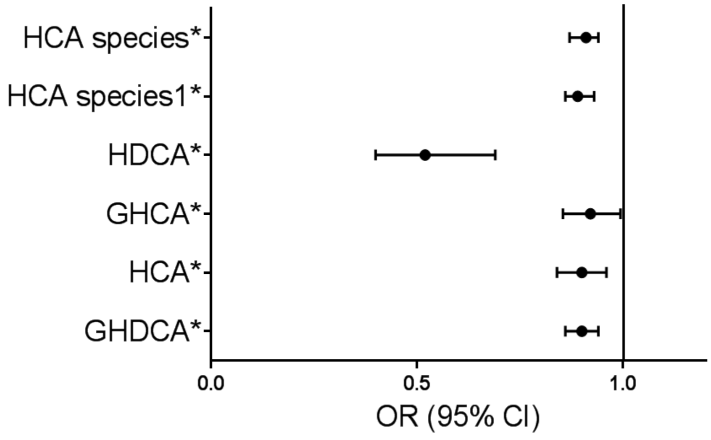

Supplementary Fig. 7. Binary logistic regression (OR with 95% CI) of total and individual HCA species indicates their potential use as risk factors for future metabolic unhealthy outcomes in the 10-year longitudinal study. HCA species1 represents the result of total HCA species after adjustment of age, sex and BMI. * p <0.05

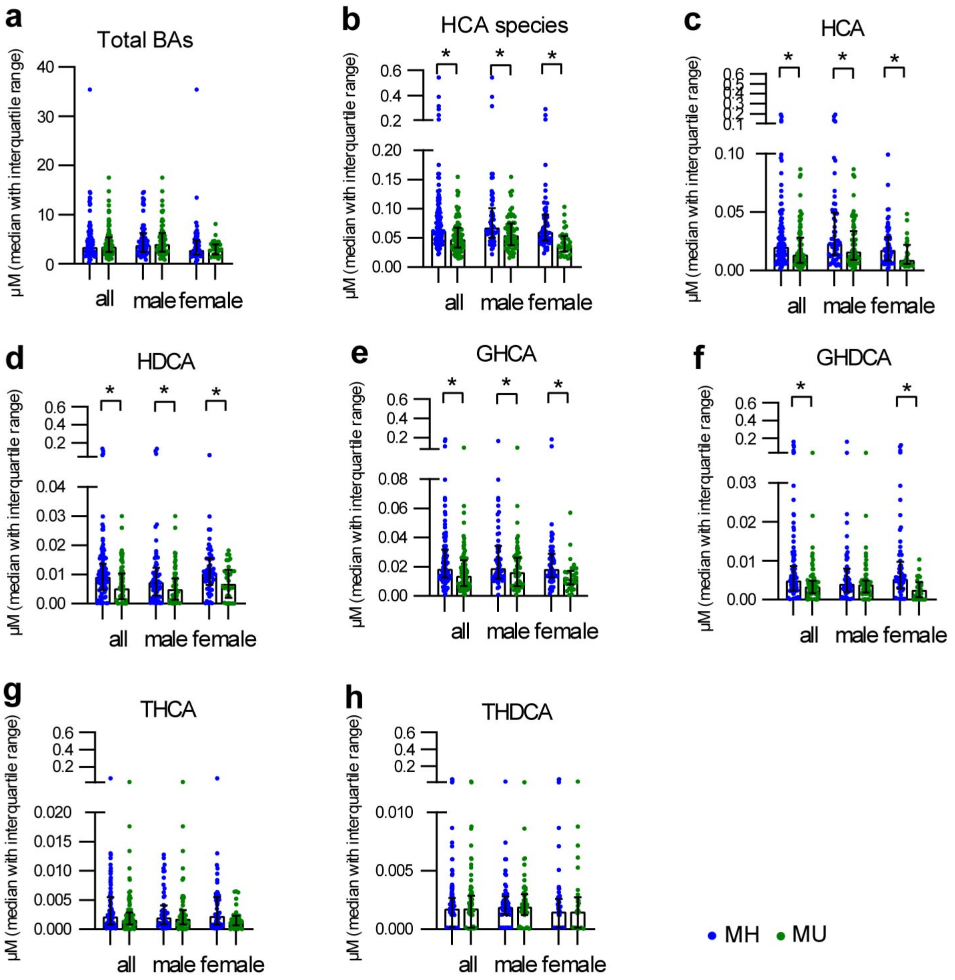

Supplementary Fig. 8. Total BAs, total and individual HCA species in the serum of all (n=207), male (n=117) and female (n=90) individuals in future metabolically healthy (MH) (n=117) and metabolically unhealthy (MU) (n=90) groups in the 5-year longitudinal study. Data are expressed as median with interquartile range. * indicate $p<0.05$ based on two-sided Mann-Whitney U test when comparing MH and MU.

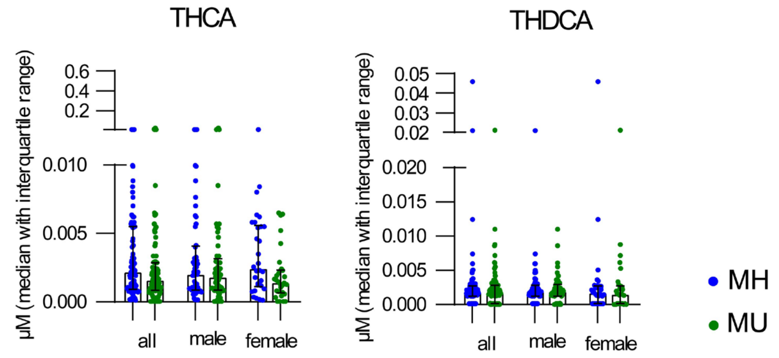

Supplementary Fig. 9. In the 5-year longitudinal study, the concentrations of THCA and THDCA in the serum of matched all (n=174), male (n=114) and female (n=60) individuals in future MH (n=87) and MU (n=87) groups. Data are expressed as median with interquartile range.

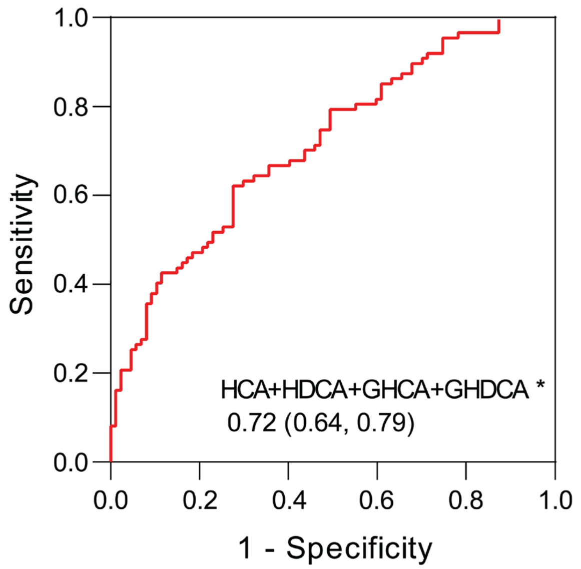

Supplementary Fig. 10. ROC analyses of the summation of HCA, HDCA, GHCA and GHDCA for the metabolic health 5-year longitudinal study (matched samples).

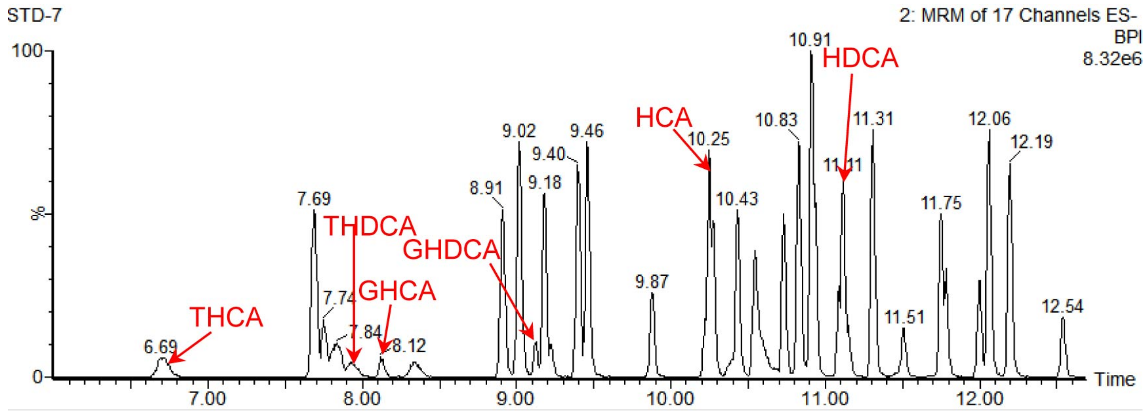

Supplementary Fig. 11. The chromatogram of TIC mode.

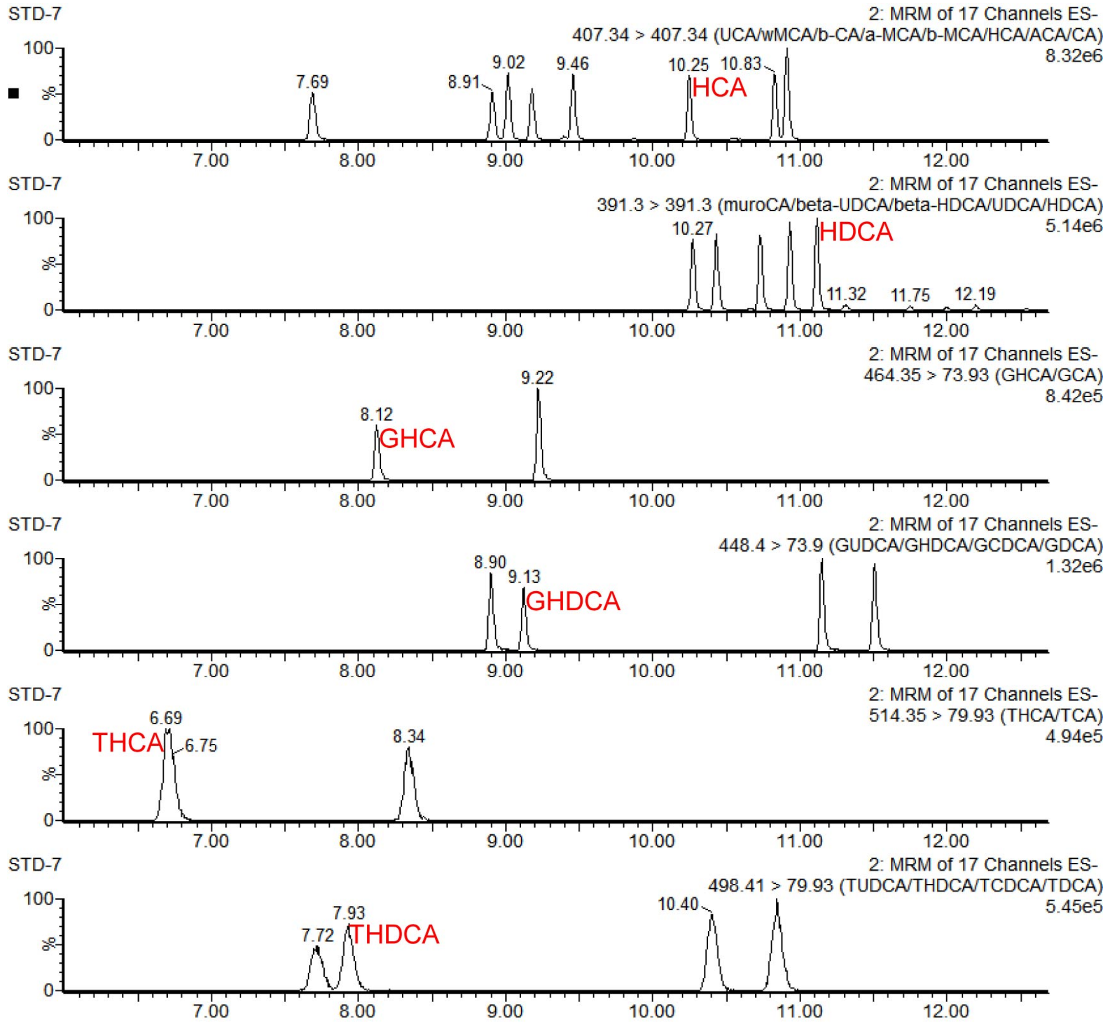

Supplementary Fig. 12. The chromatograms of MRM mode of specific channels.

Supplementary Table 1. Metabolic markers of all healthy lean (HL), healthy overweight/obese (HO) and overweight/obese with T2DM (OD) subjects in the first cross-sectional study

	Healthy lean (HL) (n=585)	Healthy overweight/obese (HO) (n=419)	Overweight/obese with T2DM (OD) (n=103)
n(M/F)	585 (329/256)	419 (229/190)	103 (52/51)
Age (year)	37±12	40±12*	53±10*#
BMI (kg/m^2)	21.34±1.79	28.28±3.37*	29.02±3.46*#
Glu0 (mmol/L)	5±0.5	5.2±0.5*	8.2±2.5*#
Glu120 (mmol/L)	5.3±0.9	6.4±1.6*	14.4±4*#
Ins0 (mU/L)	5.92±2.71	11.26±10.89*	16.4±19.36*#
Ins120 (mU/L)	30.63±19.37	61.82±59.24*	76.42±57.44*#
HbA1c (%)	5.4±0.3	5.4±0.4	7.1±1.3*#
HOMA-IR	1.29±0.63	2.63±3.02*	6.03±7.81*#
TC (mmol/L)	4.76±0.94	4.68±0.94	6.21±1.67*#
TG (mmol/L)	1.19±0.65	1.4±0.77*	2.54±2.39*#
HDL-c (mmol/L)	1.54±0.3	1.69±1.29*	1.19±0.24*#
LDL-c (mmol/L)	2.5±0.52	2.72±0.47*	3.19±0.84*#
SP (mmHg)	113±11	116±13*	138±20*#
DP (mmHg)	72±7	75±9*	83±13*#
HeartRate (bpm)	76±8	76±7	78±6*#
ALT (U/L)	24.9±14.2	30.1±17.8*	28.9±19.6
AST (U/L)	20.8±5.7	22.1±7.3*	23.4±11.5

Values were presented as number or mean ± S. D.

* Two-sided Kruskal-Wallis test $p<0.05$ when compared with HL. # Two-sided Kruskal-Wallis test $p<0.05$ when compared with HO. Chi-Square was used to compare sex ratios between groups.

BMI = body mass index; SP = systolic blood pressure; DP = diastolic blood pressure; Glu0 = fasting plasma glucose; Glu120 = 2 h plasma glucose; Ins0 = fasting insulin; Ins120 = 2 h insulin; TG = total triglycerides; TC = total cholesterol; HDL-c = high density lipoprotein cholesterol; LDL-c = low density lipoprotein cholesterol; ALT = alanine aminotransferase; AST = aspartate aminotransferase; HOMA-IR = Glu0×Ins0/22.5.

Supplementary Table 2. The levels (nM) of BA species of all healthy lean (HL), healthy overweight/obese (HO) and overweight/obese with T2DM (OD) subjects in the first cross-sectional study

	Healthy lean (HL) (n=585)	Healthy overweight/obese (HO) (n=419)	Overweight/obese with T2DM (OD) (n=103)
HCA species	36.48(26.36, 52.54)	28.45(19.96, 42.96)*	19.53(11, 34.4)*#
CA Species	167.39(81.61, 331.01)	171.33(82.01, 367.75)	151.4(86.05, 282.56)
CDCA Species	615.63(349.59, 1146.45)	731.11(390.91, 1353.44)*	684.76(386.66, 1063.29)
DCA Species	229.22(126.13, 352.59)	170.43(84.3, 296.75)*	169.03(34.52, 321.96)*
UDCA Species	85.86(47.44, 166.77)	83.95(42.4, 164.76)	128.53(52.24, 202.3)#
LCA Species	6.31(3.84, 7.81)	3.54(2.5, 7.1)	2.9(1.75, 7.3)*

Values were presented as median (first quartile, third quartile).

* Two-sided Kruskal-Wallis test *p*<0.05 when compared with HL. # Two-sided Kruskal-Wallis test *p*<0.05 when compared with HO.

HCA species = HCA+HDCA+GHCA+GHDCA, CA species=CA+GCA+TCA, CDCA species=CDCA+GCDCA+TCDCA, DCA species=DCA+GDCA+TDCA, UDCA species=UDCA+GUDCA+TUDCA

Supplementary Table 3. The levels (nM) of BA species of matched healthy lean (HL), healthy overweight/obese (HO) and overweight/obese with T2DM (OD) subjects in the first cross-sectional study

	Healthy lean (HL) (n=103)	Healthy overweight/obese (HO) (n=103)	Overweight/obese with T2DM (OD) (n=103)
HCA species	41.83(32.12, 56.11)	29.13(20.85, 46.92)*	19.53(11, 34.4)*#
CA Species	152.78(69.34, 254.14)	208.1(92.27, 454.73)	151.4(86.05, 282.56)
CDCA Species	483.33(289.12, 871.22)	823.19(482.93, 1600.49)*	684.76(386.66, 1063.29)*
DCA Species	181.23(104.18, 314.18)	188.21(79.82, 394.7)	169.03(34.52, 321.96)
UDCA Species	80.91(41.69, 172.52)	100.2(50.14, 198.3)	128.53(52.24, 202.3)
LCA Species	6.66(3.97, 7.81)	3.45(2.63, 7.1)*	2.9(1.75, 7.3)*

Values were presented as median (first quartile, third quartile).

* Two-sided Kruskal-Wallis test *p*<0.05 when compared with HL. # Two-sided Kruskal-Wallis test *p*<0.05 when compared with HO.

Supplementary Table 4. Metabolic markers of healthy control (C), pre-diabetic (PreDM) and diabetic (DM) subjects in the second cross-sectional study

	Healthy control (C) (n=26)	Pre-diabetic (PreDM) (n=30)	Diabetic (DM) (n=35)
n(M/F)	26 (9/17)	30 (10/20)	35 (16/19)
Age (year)	64±12	65±11	63±9
BMI (kg/m^2)	23.07±4.38	24.66±3.13	25.72±3.41*
Glu0 (mmol/L)	5±0.4	6.2±0.6*	8.3±4.6*
Glu120 (mmol/L)	6.7±0.6	8.4±1.2*	13.2±1.5*#
Ins0 (mU/L)	9.68±4.47	10.02±4.71	10.88±5.35
Ins120 (mU/L)	68.9±27.97	102.27±44.9	91.31±60.89
HbA1c (%)	4.9±0.6	5.8±0.5*	6.5±2*
HOMA-IR	2.3±1.03	2.4±1.15	3.34±2.02
SP (mmHg)	124±8	132±15	127±12
DP (mmHg)	90±31	78±10	77±8

Values were presented as number or mean ± S. D.

* Two-sided Kruskal-Wallis test $p<0.05$ when compared with healthy control (C). # Two-sided Kruskal-Wallis test $p<0.05$ when compared with Pre-diabetic (PreDM). Chi-Square was used to compare sex ratios between groups.

Supplementary Table 5. Serum BA concentration (nM) of healthy control (C), pre-diabetic (PreDM) and diabetic (DM) subjects in the second cross-sectional study

	Healthy control (C) (n = 26)	Pre-diabetes (PreDM) (n = 30)	Diabetes (DM) (n = 35)
HCA	15.65(9.74, 18.93)	10.40(6.25, 15.50)*	6.92(1.98, 15.20)*
HDCA	6.29(3.42, 9.23)	5.46(4.29, 6.61)	5.33(4.36, 8.42)
GHCA	11.12(8.40, 15.96)	5.03(3.17, 8.78)*	6.79(3.89, 9.15)*
GHDCA	11.82(5.83, 18.14)	8.28(3.14, 15.62)	8.33(2.18, 10)*
CA	40.44(22.35, 83.69)	57.16(26.02, 156.74)	41.69(19.83, 91.67)
GCA	180.63(78.85, 248.83)	109.81(44.67, 163.64)*	85.76(39.37, 233.15)
TCA	23.63(7.23, 37.39)	6.60(3.08, 17.72)*	12.44(2.97, 21.16)
CDCA	221.76(65.75, 359.90)	258.08(82.75, 371.76)	119.35(59.48, 435.35)
GCDCA	502.01(249.15, 808.78)	518.86(287.75, 754.24)	294.59(156.97, 578.55)#
TCDCA	37(28.99, 62.60)	24.05(8.43, 43.93)*	32.28(11.61, 50.35)
DCA	76.79(12.65, 248.53)	87.98(9.16, 197.45)	142.27(75.19, 255.26)
GDCA	79.15(39.64, 201.18)	54.88(5.47, 117.25)	98.59(40.81, 207.10)#
TDCA	17.39(8.14, 36.69)	7.80(3.84, 12.65)*	21.02(5.45, 31.13)#
UDCA	80.71(15.56, 138.99)	68.06(35.79, 142.05)	49.21(21.63, 125.10)
GUDCA	64.91(24.17, 126)	35.46(18.06, 63.29)	32.88(13.11, 62.43)*
TUDCA	3.39(1.03, 5.52)	1.02(0.50, 3.09)*	1.45(0.77, 2.88)*
GLCA	1.88(0.27, 5.32)	1.34(0.25, 4.09)	3.83(0.62, 9.54)#
TLCA	2.12(1.57, 3.06)	1.59(1.46, 2.04)	2.93(1.66, 4.49)#
NorDCA	1.98(1.25, 2.62)	2.51(1.99, 2.69)	2.13(1.75, 2.51)
7-ketoLCA	8.40(3.81, 16.40)	7.30(2.10, 14.41)	5.58(2.27, 16.56)
7-ketoDCA	3.33(2.89, 5.62)	3.74(3.08, 5.38)	3.38(2.84, 5.28)
12-ketoCDCA	21.90(2.81, 47.34)	11.28(1.80, 29.75)	16.92(5.01, 49.28)
3-ketoCA	2.94(1.52, 5.85)	3.83(1.57, 8.47)	1.85(0.87, 3.75)#

Values were presented as median (first quartile, third quartile).

* Two-sided Kruskal-Wallis test $p<0.05$ when compared with C. # Two-sided Kruskal-Wallis test $p<0.05$ when compared with PreDM.

Supplementary Table 6. Fecal BA concentration (nmol/g) of healthy control (C), pre-diabetic (PreDM) and diabetic (DM) subjects in the second cross-sectional study

	Healthy control (C) (n = 26)	Pre-diabetes (PreDM) (n = 30)	Diabetes (DM) (n = 35)
HCA	8.97(5.34, 10.33)	3.11(0.33, 7.02)*	1.59(0.51, 5.86)*
HDCA	6.47(6.09, 9.31)	3.72(1.31, 5.79)*	2.26(1.25, 4.98)*
CA	67.04(7.29, 2382.11)	76.68(15.44, 1611.53)	24(11.86, 1385.19)
GCA	5.38(2.26, 9.97)	8.03(3.73, 23.62)	4.52(1.69, 14.23)
TCA	2.35(0.41, 10.29)	2.16(0.47, 12.27)	2.52(1.22, 11.35)
CDCA	49.73(9.61, 364.44)	97.13(11.3, 569.85)	35.53(10.56, 209.86)
GCDCA	10.41(5.53, 17.10)	18.30(6.21, 41.72)	11.03(3.14, 21.93)
DCA	616.69(60.27, 928.9)	411.97(156.84, 1044.42)	697.62(315.80, 1117.51)
GDCA	7.14(0.72, 14.87)	5.35(0.87, 16.29)	9.98(2.78, 17.43)
UDCA	25.23(4.14, 481.33)	150.95(7.56, 1545.87)	33.39(11.20, 452.01)
GUDCA	1.30(0.27, 3.98)	3.05(0.37, 8.25)	1.12(0.13, 2.63)#
LCA	2136.56(753.45, 2757.16)	1883.91(54.09, 2881.24)	2290.13(1321.26, 3225.10)
GLCA	0.42(0.11, 1.63)	0.34(0.02, 1.42)	0.65(0.07, 1.45)
TLCA	1.75(1.04, 3.14)	1.63(0.93, 2.74)	1.47(0.97, 2.25)
12-ketoCDCA	0.63(0.15, 1.30)	0.53(0.28, 0.66)	0.60(0.22, 0.98)
3-ketoCA	1.31(0.32, 75.68)	1.35(0.54, 143.14)	0.77(0.36, 15.67)
7-ketoDCA	10.17(0.98, 128.30)	15.83(1.06, 389.84)	3.27(0.79, 78.97)
7-ketoLCA	81.29(1.87, 403.75)	159.90(7.47, 1015.89)	28.59(6.18, 156.86)
NorDCA	0.07(0.03, 0.22)	0.17(0.04, 0.32)	0.12(0.04, 0.46)

Values were presented as median (first quartile, third quartile).

* Two-sided Kruskal-Wallis test $p<0.05$ when compared with C. # Two-sided Kruskal-Wallis test $p<0.05$ when compared with PreDM.

Supplementary Table 7. Serum BA concentration (nM) of individuals with diabetes at baseline (0m) and 1, 3, 6, and 12 months after gastric bypass surgery

	Baseline (0m)	1 month after	3 month after	6 month after	12 months after
HCA	3.92(1.56, 10.71)	6.35(2.35, 27.06)*	15.70(4.79, 32.07)*	10.52(6.04, 31.76)*	10.85(3.14, 35.37)*
HDCA	2.26(1.21, 3.86)	5.54(2.90, 17.04)*	5.84(2.06, 21.93)*	10.86(5.61, 23.14)*	11.04(1.89, 17.35)*
GHCA	2.67(1.45, 5.27)	6.86(3.07, 20.61)*	11.54(2.76, 18.34)*	11.71(4.59, 19.73)*	5.06(2.30, 18.63)*
GHDCA	3.71(0.60, 7.78)	9.40(1.87, 20.85)*	18.19(6.42, 23.73)*	9.79(4.07, 19.78)*	10.05(3.59, 27.95)*
CA	17.34(10.15, 58.36)	15.28(6.49, 47.86)	22.44(12.89, 255.93)	18.78(11.21, 69.55)	20.69(8.91, 164.63)
GCA	108.28(48.52, 195.25)	104.81(49.34, 224.18)	130.78(81.20, 250.23)	168.71(87.12, 297.22)	109.98(61.01, 300.76)
TCA	8.42(4.05, 17.88)	17.48(7.56, 34.18)	13.24(5.52, 27.30)	13.98(4.86, 27.05)	5.80(3.65, 20.15)
CDCA	240.77(108.33, 404.50)	53.98(15.14, 158.66)*	49.44(15.34, 240.95)*	49.84(29.08, 188.39)*	130.21(38.41, 205.34)*
GCDCA	360.39(123.57, 567.82)	364.22(117.36, 733.83)	396.05(180.11, 769.01)	416(148.31, 669.62)	575.49(287.92, 1093.65)*
TCDCA	18.45(9.97, 51.18)	35.34(19.35, 114.99)*	29.09(7.08, 77.32)	35.50(12.23, 59.33)	20.20(7.66, 44.24)
DCA	111.28(17.15, 227.36)	54.73(10.89, 103.99)*	94.41(34.59, 231.29)	91.68(49.39, 171.75)	154.06(72.68, 281.47)
GDCA	27.90(9.13, 69.90)	21.13(8.15, 72.03)	47.40(20.73, 105.37)	46.44(21.06, 89.92)	39.14(16.44, 110.99)
TDCA	6.02(3.26, 12.03)	10.72(3.47, 21.74)	9.29(3.24, 19.30)	8.13(4.74, 24.23)	7.65(4.38, 19.01)
UDCA	60.89(23.03, 187.89)	35.42(5.32, 102.45)*	29.68(9.64, 131.87)	27.61(13.03, 104.32)*	44.63(19.54, 119.54)
GUDCA	14.78(0.26, 48.27)	0.26(0.26, 44.66)	0.26(0.26, 28.17)*	0.26(0.26, 17.76)*	0.26(0.26, 18.63)*
TUDCA	3.49(3.06, 4.88)	4.48(3, 8.20)	3.37(1.54, 5.79)	3.33(0.28, 4.51)	3.05(0.28, 4.36)*
GLCA	3.34(0.30, 5.73)	3.35(0.30, 5.45)	3.67(0.30, 8.07)	4.23(3.16, 6.22)	4.62(3.11, 11.78)*
TLCA	0.51(0.51, 5.80)	0.51(0.51, 6.31)	0.51(0.51, 0.51)	0.51(0.51, 6.01)	0.51(0.51, 6.35)
NorDCA	1.55(0.12, 2.23)	1.97(1.33, 3.81)*	2.37(1.34, 3.92)*	1.94(1.34, 3.34)	1.85(0.12, 3.44)
7-ketoLCA	12.61(6.21, 19.20)	4.45(1.93, 15.29)*	6.57(2.14, 17.44)	3.81(1.35, 11.69)*	5.51(2.30, 19.21)*
7-ketoDCA	5.10(0.18, 11.43)	1.85(0.18, 8.52)	2.76(1.91, 13.25)	2.35(0.18, 4.57)*	3.40(0.18, 8.64)
12-ketoCDCA	4.84(0.87, 17.16)	15.52(0.87, 39.99)*	13.38(0.87, 30.05)	11.33(0.87, 33.59)	12.73(0.87, 24.96)
3-ketoCA	18.17(10.87, 29.73)	14.42(0.92, 31.18)	17.48(9.93, 30.86)	13.75(0.92, 18.39)	12.97(0.92, 32.25)

Values were presented as median (first quartile, third quartile).

* *p*<0.05 when compared with baseline (0m) based on two-sided Wilcoxon paired samples signed-rank test. N=38 at each time point.

Supplementary Table 8. Baseline metabolic markers of patients with diabetes in the remission and non-remission groups in the gastric bypass surgery intervention study

	remission (n = 26)	non-remissssion (n = 12)
n(M/F)	26 (13/13)	12 (5/7)
Age (year)	44±13	48±12
BMI (kg/m^2)	32.76±3.92	31.02±3.51
Waist (cm)	109±14	103±9
Glu0 (mmol/L)	7.4±2	9.4±3*
Glu120 (mmol/L)	11.6±4.1	14±3.3
Ins0 (mU/L)	23.49±18.13	29.83±30.11
Ins120 (mU/L)	117.29±81.84	79.12±68.91
TC (mmol/L)	5.03±0.19	5.52±0.32
TG (mmol/L)	2.00±0.24	3.99±1.62
HDL-c (mmol/L)	1.03±0.13	0.99±0.22
LDL-c (mmol/L)	3.07±0.86	3.16±1.03
HbA1c (%)	7.2±1.7	8.7±1.3*
HOMA-IR	7.56±5.51	11.87±10.67

Values were presented as number or mean ± S. D.

* Two-sided Mann-Whitney U test $p<0.05$ when comparing the two groups. Chi-Square was used to compare sex ratio between groups.

Supplementary Table 9. Baseline serum BA concentration (nM) of individuals with diabetes in the remission and non-remission groups in the gastric bypass surgery intervention study

	remission (n = 26)	non-remission (n = 12)
HCA	6.34(2.01, 12.45)	1.49(0.72, 4.07)*
HDCA	2.42(1.41, 4.49)	1.71(0.83, 3.85)
GHCA	3.28(1.79, 13.18)	0.89(0.25, 3.12)*
GHDCA	5.09(0.64, 13.73)	0.25(0.25, 4.55)*
CA	24.71(14.97, 126.53)	10.29(8.77, 14.83)*
GCA	122.84(90.34, 215.82)	73.97(42.47, 155.36)
TCA	10.71(4.83, 20.02)	7.5(3.55, 13.47)
CDCA	269.91(95.98, 573.53)	239.3(135.68, 332.19)
GCDCA	213.66(90.85, 480.24)	581.64(330.71, 988.04)*
TCDCA	19.76(10.25, 49.35)	13.23(7.3, 54.97)
DCA	156.89(55.47, 254.94)	29.81(0.06, 198.62)
GDCA	31.09(19.87, 71.34)	16.38(0.2, 51.06)
TDCA	7.13(3.29, 12.03)	4.09(0.31, 18.37)
UDCA	60.89(24.87, 222.7)	64.86(20.44, 179.35)
GUDCA	7.56(0.26, 48.27)	24.34(7.48, 48.04)
TUDCA	3.50(2.98, 6.14)	3.49(3.1, 4.22)
GLCA	3.34(0.3, 4.55)	3.65(0.3, 6.31)
TLCA	0.51(0.51, 5.8)	0.51(0.51, 6.58)
12-ketoCDCA	9.56(0.87, 19.28)	0.87(0.87, 13.28)
3-ketoCA	18.17(11.29, 31.73)	18.11(3.41, 29.07)
7-ketoDCA	6.60(0.18, 12.35)	3.51(0.18, 11.03)
7-ketoLCA	14.31(8.42, 21.61)	7.42(2.39, 18)
NorDCA	1.59(0.94, 2.31)	1.39(0.12, 1.89)

Values were presented as median (first quartile, third quartile).

* Two-sided Mann-Whitney U test $p<0.05$ when comparing the two groups.

Supplementary Table 10. Baseline metabolic markers of all future metabolically healthy (MH) and unhealthy (MU) groups in the 10-year longitudinal study

	future metabolically healthy (MH)	future metabolically unhealthy (MU)
n(M/F)	46 (10/36)	86 (26/60)
Age (year)	33±10	40±12*
BMI (kg/m^2)	22.96±2.82	24.6±3.49*
Waist (cm)	74.11±9.04	79.68±9.94*
Glu0 (mmol/L)	4.7±0.4	4.7±0.4
Glu120 (mmol/L)	4.6±0.9	5.1±1.1
Ins0 (mU/L)	6.29±2.73	6.59±3.21
Ins120 (mU/L)	31.34±25	34.83±22.45
HDL-c (mmol/L)	1.36±0.2	1.35±0.18
LDL-c (mmol/L)	2.52±0.53	2.68±0.48
SP (mmHg)	109±12	113±12
DP (mmHg)	71±6	73±6
HOMA-IR	1.33±0.63	1.38±0.71

Values were presented as mean ± S. D.

* Two-sided Mann-Whitney U test $p<0.05$ when comparing the two groups. Chi-Square was used to compare sex ratio between groups.

Supplementary Table 11. Baseline metabolic markers of matched future metabolically healthy (MH) and unhealthy (MU) groups in the 10-year longitudinal study

	future metabolically healthy (MH)	future metabolically unhealthy (MU)
n(M/F)	46 (10/36)	46 (10/36)
Age (year)	33±10	33±6
BMI (kg/m^2)	22.96±2.82	22.31±2.12
Waist (cm)	74.11±9.04	74.11±7.32
Glu0 (mmol/L)	4.7±0.4	4.6±0.4
Glu120 (mmol/L)	4.6±0.9	4.9±1.1
Ins0 (mU/L)	6.29±2.73	6.26±2.87
Ins120 (mU/L)	31.34±25	31.82±21.3
HDL-c (mmol/L)	1.36±0.2	1.37±0.17
LDL-c (mmol/L)	2.52±0.53	2.64±0.53
SP (mmHg)	109±12	109±11
DP (mmHg)	71±6	72±7
HOMA-IR	1.33±0.63	1.3±0.65

Values were presented as mean ± S. D.

Supplementary Table 12. Baseline serum BA concentration (nM) of all future metabolically healthy (MH) and unhealthy (MU) groups in the 10-year longitudinal study

	future metabolically healthy (MH) (n=46)	future metabolically unhealthy (MU) (n=86)
HCA	15.10(6.10, 31.02)	11.04(4.24, 17.46)*
HDCA	9.48(7.05, 11.24)	4.27(2.04, 5.69)*
GHCA	7.39(3.72, 16.18)	4.61(1.72, 8.41)*
GHDCA	20.50(15.51, 24.15)	8.60(6.17, 11.33)*
CA	65.57(20.88, 129.40)	55.49(16.61, 141.69)
GCA	110.86(64.29, 219.61)	100.94(56.20, 223.14)
TCA	5.55(2.50, 18.85)	8.98(5.27, 16.73)
CDCA	213.43(70.77, 383.54)	160.59(72.69, 340.76)
GCDCA	388.14(221.49, 596.63)	350.28(227.90, 602.38)
TCDCA	23.90(11.81, 41.58)	22.58(11.50, 41.07)
DCA	115.70(76.79, 210.25)	122.02(50.68, 197.11)
GDCA	67.47(29.49, 104.89)	61.17(25.90, 120.12)
TDCA	6.50(1.41, 14.93)	4.69(1.05, 17.34)
UDCA	52.94(18.56, 96.50)	62.96(26.30, 109.38)
GUDCA	34.80(22.97, 58.71)	30.16(16.18, 70.39)
TUDCA	1.33(0.72, 2.78)	1.22(0.70, 4.05)
GLCA	0.60(0.21, 4.15)	0.59(0.19, 4.43)
TLCA	0.95(0.66, 1.67)	0.79(0.61, 1.24)
12-ketoCDCA	10.33(3.77, 29.29)	8.03(2.94, 23.08)
3-ketoCA	0.72(0.61, 2.71)	1.36(0.68, 2.76)
7-ketoDCA	1.85(0.80, 11.57)	2.20(0.83, 4.10)
7-ketoLCA	5.69(2.21, 13.68)	6.75(2.36, 12.90)
NorDCA	2.09(1.25, 3.28)	1.46(0.76, 1.72)*

Values were presented as median (first quartile, third quartile).

* Two-sided Mann-Whitney U test $p<0.05$ when comparing the two groups.

Supplementary Table 13. Baseline serum BA concentration (nM) of matched future metabolically healthy (MH) and unhealthy (MU) groups in the 10-year longitudinal study

	future metabolically healthy (MH) (n=46)	future metabolically unhealthy (MU) (n=46)
HCA	15.10(6.10, 31.02)	9.99(4.15, 13.62)*
HDCA	9.48(7.05, 11.24)	4.45(2.41, 5.18)*
GHCA	7.39(3.72, 16.18)	4.36(1.67, 8.95)*
GHDCA	20.50(15.51, 24.15)	8.24(4.38, 11.33)*
CA	65.57(20.88, 129.40)	54.79(16.27, 128.50)
GCA	110.86(64.29, 219.61)	109.19(48.01, 226.87)
TCA	5.55(2.50, 18.85)	10.56(6.48, 16.81)
CDCA	213.43(70.77, 383.54)	164.29(69.84, 410.08)
GCDCA	388.14(221.49, 596.63)	377.12(227.90, 600.04)
TCDCA	23.90(11.81, 41.58)	20.90(11.50, 41.07)
DCA	115.70(76.79, 210.25)	112.71(36.15, 203.18)
GDCA	67.47(29.49, 104.89)	60.96(23.22, 114.42)
TDCA	6.50(1.41, 14.93)	6.34(0.92, 18.31)
UDCA	52.94(18.56, 96.50)	62.96(30.45, 114.93)
GUDCA	34.80(22.97, 58.71)	29.88(16.18, 91.55)
TUDCA	1.33(0.72, 2.78)	1.24(0.72, 4.44)
GLCA	0.60(0.21, 4.15)	0.52(0.16, 4.65)
TLCA	0.95(0.66, 1.67)	0.79(0.66, 1.05)
12-ketoCDCA	10.33(3.77, 29.29)	5.06(2.91, 18.52)
3-ketoCA	0.72(0.61, 2.71)	1.00(0.63, 3.16)
7-ketoDCA	1.85(0.80, 11.57)	2.21(1.24, 4.03)
7-ketoLCA	5.69(2.21, 13.68)	6.91(2.51, 13.87)
NorDCA	2.09(1.25, 3.28)	0.76(0.76, 1.48)*

Values were presented as median (first quartile, third quartile).

* Two-sided Mann-Whitney U test $p<0.05$ when comparing the two groups.

Supplementary Table 14. Baseline metabolic markers of all future metabolically healthy and unhealthy groups in the 5-year longitudinal study

	future metabolically healthy (MH)	future metabolically unhealthy (MU)
n(M/F)	117 (57/60)	90 (60/30)
Age (year)	39±10	40±10
BMI (kg/m^2)	22.5±2.56	23.11±2.48*
Waist (cm)	77.53±8.55	79.77±7.68
Glu0 (mmol/L)	5.3±0.4	5.4±0.3
HDL-c (mmol/L)	1.32±0.3	1.3±0.29
LDL-c (mmol/L)	2.5±0.53	2.63±0.6
SP (mmHg)	119±10	120±10
DP (mmHg)	73±6	74±8*
TG (mmol/L)	0.99±0.3	1.04±0.28
TC (mmol/L)	4.59±0.69	4.7±0.77

Values were presented as mean ± S. D.

* Two-sided Mann-Whitney U test p<0.05 when comparing the two groups. Chi-Square was used to compare sex ratio between groups.

Supplementary Table 15. Baseline metabolic markers of matched future metabolically healthy and unhealthy groups in the 5-year longitudinal study

	future metabolically healthy (MH)	future metabolically unhealthy (MU)
n(M/F)	87 (57/30)	87 (57/30)
Age (year)	40±10	40±10
BMI (kg/m^2)	22.41±2.48	23.08±2.48
Waist (cm)	79±8	80±8
Glu0 (mmol/L)	5.3±0.4	5.4±0.3
HDL-c (mmol/L)	1.31±0.29	1.3±0.29
LDL-c (mmol/L)	2.51±0.53	2.64±0.6
SP (mmHg)	120±10	120±11
DP (mmHg)	73±6	75±8
TG (mmol/L)	0.99±0.31	1.03±0.28
TC (mmol/L)	4.6±0.69	4.7±0.78

Values were presented as mean ± S. D.

Supplementary Table 16. Baseline serum BA concentration (nM) of all future metabolically healthy (MH) and unhealthy (MU) groups in the 5-year longitudinal study

	future metabolically healthy (MH) (n=117)	future metabolically unhealthy (MU) (n=90)
HCA	21.23(12.37, 39.02)	13.38(6.58, 26.62)*
HDCA	9.20(4.66, 13.42)	5.33(1.18, 10.3)*
GHCA	21.10(12.66, 32.78)	13.70(6.84, 24.38)*
GHDCA	4.65(2.31, 8.5)	3.23(1.61, 4.6)*
THCA	2.19(0.94, 5.5)	1.60(0.83, 2.88)
THDCA	1.83(1.22, 2.72)	1.67(0.13, 2.84)
CA	105.03(44.79, 259.43)	59.50(29.68, 236.45)*
GCA	154.70(80.6, 284.97)	125.43(57.32, 323.91)
TCA	14.08(6.94, 21.57)	12.10(6.6, 25.6)
CDCA	414.00(218.64, 892.4)	485.74(161.04, 833.87)
GCDCA	1473.52(862.53, 2491)	1447.90(790.4, 2526.35)
TCDCA	80.40(46.04, 152.63)	71.10(42.73, 165.34)
DCA	380.38(213.1, 574.66)	270.18(127.35, 482.32)
GDCA	242.88(111.77, 519.57)	154.00(67.96, 280.66)*
TDCA	20.60(6.24, 46.1)	15.73(6.8, 30.07)
UDCA	88.21(36.51, 218.69)	84.39(34.43, 174.42)
GUDCA	167.69(84.73, 318.29)	143.3(68.17, 306.42)
TUDCA	2.79(0.35, 5.3)	2.10(0.74, 6.2)
GLCA	13.00(3.49, 25.8)	9.73(1.31, 21.56)
TLCA	1.20(0.69, 3.18)	1.17(0.65, 3.01)
6,7-diketoLCA	0.33(0.18, 0.9)	0.23(0.15, 0.9)
7,12-diketoLCA	0.94(0.36, 1.5)	1.13(0.35, 1.68)
7-DHCA	4.16(1.84, 8.36)	3.17(1.8, 7)
bCA	1.48(0.27, 4.52)	1.69(0.2, 5.12)
muroCA	1.55(0.66, 3.11)	1.55(0.67, 2.5)
NorDCA	1.59(0.59, 3.28)	0.94(0.17, 2.31)
UCA	1.90(1.13, 3.16)	1.70(0.5, 2.5)

Values were presented as median (first quartile, third quartile).

* Two-sided Mann-Whitney U test $p<0.05$ when comparing the two groups.

Supplementary Table 17. Baseline serum BA concentration (nM) of matched future metabolically healthy (MH) and unhealthy (MU) groups in the 5-year longitudinal study

	future metabolically healthy (MH) (n=87)	future metabolically unhealthy (MU) (n=87)
HCA	21.23(12.37, 39.02)	13.38(6.58, 26.62)*
HDCA	9.20(4.66, 13.42)	5.33(1.18, 10.3)*
GHCA	21.10(12.66, 32.78)	13.70(6.84, 24.38)*
GHDCA	4.65(2.31, 8.5)	3.23(1.61, 4.6)*
THCA	2.19(0.94, 5.5)	1.60(0.83, 2.88)
THDCA	1.83(1.22, 2.72)	1.67(0.13, 2.84)
CA	105.03(44.79, 259.43)	59.50(29.68, 236.45)*
CDCA	154.70(80.6, 284.97)	125.43(57.32, 323.91)
DCA	14.08(6.94, 21.57)	12.10(6.6, 25.6)
UDCA	414.00(218.64, 892.4)	485.74(161.04, 833.87)
NorDCA	1473.52(862.53, 2491)	1447.90(790.4, 2526.35)
TCA	80.40(46.04, 152.63)	71.10(42.73, 165.34)
TCDCA	380.38(213.1, 574.66)	270.18(127.35, 482.32)
TDCA	242.88(111.77, 519.57)	154.00(67.96, 280.66)*
TLCA	20.60(6.24, 46.1)	15.73(6.8, 30.07)
GCA	88.21(36.51, 218.69)	84.39(34.43, 174.42)
GCDCA	167.69(84.73, 318.29)	143.3(68.17, 306.42)
GDCA	2.79(0.35, 5.3)	2.10(0.74, 6.2)
GLCA	13.00(3.49, 25.8)	9.73(1.31, 21.56)
GUDCA	1.20(0.69, 3.18)	1.17(0.65, 3.01)
TUDCA	0.33(0.18, 0.9)	0.23(0.15, 0.9)
7-DHCA	0.94(0.36, 1.5)	1.13(0.35, 1.68)
6,7-diketoLCA	4.16(1.84, 8.36)	3.17(1.8, 7)
7,12-diketoLCA	1.48(0.27, 4.52)	1.69(0.2, 5.12)
muroCA	1.55(0.66, 3.11)	1.55(0.67, 2.5)
bCA	1.59(0.59, 3.28)	0.94(0.17, 2.31)
UCA	1.90(1.13, 3.16)	1.70(0.5, 2.5)

Values were presented as median (first quartile, third quartile).

* Two-sided Mann-Whitney U test $p<0.05$ when comparing the two groups.

Supplementary Table 18. The precursor-to-product ion pairs of detected BAs

Metabolites	Transition (m/z)
HCA	407.3>407.3
HDCA	391.3>391.3
GHCA	464.3>73.9
GHDCA	448.3>73.9
THCA	514.4>79.9
THDCA	498.4>79.9
CA	407.3>407.3
GCA	464.3>73.9
TCA	514.4>79.9
CDCA	391.3>391.3
GCDCA	448.3>73.9
TCDCA	498.4>79.9
DCA	391.3>391.3
GDCA	448.3>73.9
TDCA	498.4>79.9
UDCA	391.3>391.3
GUDCA	448.3>73.9
TUDCA	498.4>79.9
GLCA	432.4>73.9
12-ketoCDCA	405.4>405.4
3-ketoCA	405.4>405.4
7-ketoDCA	405.4>405.4
7-ketoLCA	389.3>389.3
NorDCA	377.3>377.3
UCA	407.3>407.3
DHCA	401.4>401.4

Supplementary Table 19. The LOD and LOQ for BA assays in Shanghai (Lab1)

	LOD (nM)	LOQ (nM)
HCA	0.20	0.50
HDCA	0.05	0.10
GHCA	0.20	0.50
GHDCA	0.20	0.50
THCA	0.20	0.50
THDCA	0.20	0.50

Supplementary Table 20. The LOD and LOQ for BA assays in Shanghai (Lab2)

	LOD (nM)	LOQ (nM)
HCA	0.10	0.20
HDCA	0.05	0.10
GHCA	0.05	0.10
GHDCA	0.05	0.10
THCA	0.05	0.10
THDCA	0.05	0.10

Supplementary Table 21. Cross-validation of methods from Lab1 and Lab2 for BA quantification using commercially available standard human plasma

	HCA		HDCA		GHCA		GHDCA		THCA		THDCA	
	Lab 1	Lab 2	Lab 1	Lab 2	Lab 1	Lab 2	Lab 1	Lab 2	Lab 1	Lab 2	Lab 1	Lab 2
Mean value of quantitation (nM) (n=6)	9.62	7.46	3.56	2.96	26.74	31.30	7.76	8.40	2.21	2.28	3.17	2.57
Intra-batch precision CV %	11.73	3.64	13.19	12.68	12.51	9.67	14.08	12.44	14.92	14.44	14.52	17.16
Inter-batch precision CV %	16.08		15.73		13.32		13.27		14.07		18.06	
