# Supplementary material for: Hyocholic acid species as novel biomarkers for metabolic disorders
Source: Nat Commun. 2021 Mar 5;12:1487. doi: 10.1038/s41467-021-21744-w (PMC7935989; doi:10.1038/s41467-021-21744-w)
Supplement: Supplementary file 2 — Reporting Summary [file 41467_2021_21744_MOESM2_ESM.pdf]

nature research

Corresponding author(s): Wei Jia

Last updated by author(s): Jan 26, 2021

Reporting Summary

Nature Research wishes to improve the reproducibility of the work that we publish. This form provides structure for consistency and transparency in reporting. For further information on Nature Research policies, see Authors & Referees and the Editorial Policy Checklist .

Statistics

For all statistical analyses, confirm that the following items are present in the figure legend, table legend, main text, or Methods section.

n/a	Confirmed	
☐	☒	The exact sample size (*n*) for each experimental group/condition, given as a discrete number and unit of measurement
☐	☒	A statement on whether measurements were taken from distinct samples or whether the same sample was measured repeatedly
☐	☒	The statistical test(s) used AND whether they are one- or two-sided<br>*Only common tests should be described solely by name; describe more complex techniques in the Methods section.*
☐	☒	A description of all covariates tested
☐	☒	A description of any assumptions or corrections, such as tests of normality and adjustment for multiple comparisons
☐	☒	A full description of the statistical parameters including central tendency (e.g. means) or other basic estimates (e.g. regression coefficient) AND variation (e.g. standard deviation) or associated estimates of uncertainty (e.g. confidence intervals)
☐	☒	For null hypothesis testing, the test statistic (e.g. *F*, *t*, *r*) with confidence intervals, effect sizes, degrees of freedom and *P* value noted<br>*Give P values as exact values whenever suitable.*
☒	☐	For Bayesian analysis, information on the choice of priors and Markov chain Monte Carlo settings
☒	☐	For hierarchical and complex designs, identification of the appropriate level for tests and full reporting of outcomes
☐	☒	Estimates of effect sizes (e.g. Cohen's *d*, Pearson's *r*), indicating how they were calculated

Our web collection on statistics for biologists contains articles on many of the points above.

Software and code

Policy information about availability of computer code

Data collection	MassLynx v4.1 and TargetLynex v4.1 (Waters, Milford, MA) were used for data collection.
Data analysis	SPSS (V19, IBM, USA), GraphPad Prism (6.0, Graphpad, USA), and IP4M 2.0 were used for statistical analyses.

For manuscripts utilizing custom algorithms or software that are central to the research but not yet described in published literature, software must be made available to editors/reviewers. We strongly encourage code deposition in a community repository (e.g. GitHub). See the Nature Research guidelines for submitting code & software for further information.

Data

Policy information about availability of data

All manuscripts must include a data availability statement. This statement should provide the following information, where applicable:

- Accession codes, unique identifiers, or web links for publicly available datasets
- A list of figures that have associated raw data
- A description of any restrictions on data availability

The source data underlying figures and tables are provided as a Source Data file. The metabolomics data were deposited and available at Metabolights repository with accession code MTBLS2343. Other data supporting the findings of this study are available from the corresponding authors upon reasonable request.

Field-specific reporting

Please select the one below that is the best fit for your research. If you are not sure, read the appropriate sections before making your selection.

☒ Life sciences ☐ Behavioural & social sciences ☐ Ecological, evolutionary & environmental sciences

Life sciences study design

All studies must disclose on these points even when the disclosure is negative.

Sample size	The sample sizes were predetermined by IP4M 2.0 (http://ip4m.cn). The power was set as 0.85 (significant level 0.05, two tails) and the effect size was set as 0.5.
Data exclusions	No data were excluded from the analyses.
Replication	The sample anaylses of the 5 cohorts were performed independently.
Randomization	Human samples were allocated into groups according to clinical diagnostic criteria. Detailed definitions and descriptions were provided in the manuscript.
Blinding	The investigators were blinded to the group allocation during data collection and pretreatment. The instrument running order was randomized. Investigators knew the group allocation after raw data pretreatment for statistical analysis.

Behavioural & social sciences study design

All studies must disclose on these points even when the disclosure is negative.

Study description	*Briefly describe the study type including whether data are quantitative, qualitative, or mixed-methods (e.g. qualitative cross-sectional, quantitative experimental, mixed-methods case study).*
Research sample	*State the research sample (e.g. Harvard university undergraduates, villagers in rural India) and provide relevant demographic information (e.g. age, sex) and indicate whether the sample is representative. Provide a rationale for the study sample chosen. For studies involving existing datasets, please describe the dataset and source.*
Sampling strategy	*Describe the sampling procedure (e.g. random, snowball, stratified, convenience). Describe the statistical methods that were used to predetermine sample size OR if no sample-size calculation was performed, describe how sample sizes were chosen and provide a rationale for why these sample sizes are sufficient. For qualitative data, please indicate whether data saturation was considered, and what criteria were used to decide that no further sampling was needed.*
Data collection	*Provide details about the data collection procedure, including the instruments or devices used to record the data (e.g. pen and paper, computer, eye tracker, video or audio equipment) whether anyone was present besides the participant(s) and the researcher, and whether the researcher was blind to experimental condition and/or the study hypothesis during data collection.*
Timing	*Indicate the start and stop dates of data collection. If there is a gap between collection periods, state the dates for each sample cohort.*
Data exclusions	*If no data were excluded from the analyses, state so OR if data were excluded, provide the exact number of exclusions and the rationale behind them, indicating whether exclusion criteria were pre-established.*
Non-participation	*State how many participants dropped out/declined participation and the reason(s) given OR provide response rate OR state that no participants dropped out/declined participation.*
Randomization	*If participants were not allocated into experimental groups, state so OR describe how participants were allocated to groups, and if allocation was not random, describe how covariates were controlled.*

Ecological, evolutionary & environmental sciences study design

All studies must disclose on these points even when the disclosure is negative.

Study description	*Briefly describe the study. For quantitative data include treatment factors and interactions, design structure (e.g. factorial, nested, hierarchical), nature and number of experimental units and replicates.*
Research sample	*Describe the research sample (e.g. a group of tagged Passer domesticus, all Stenocereus thurberi within Organ Pipe Cactus National Monument), and provide a rationale for the sample choice. When relevant, describe the organism taxa, source, sex, age range and any manipulations. State what population the sample is meant to represent when applicable. For studies involving existing datasets, describe the data and its source.*
Sampling strategy	*Note the sampling procedure. Describe the statistical methods that were used to predetermine sample size OR if no sample-size calculation was performed, describe how sample sizes were chosen and provide a rationale for why these sample sizes are sufficient.*
Data collection	*Describe the data collection procedure, including who recorded the data and how.*

Timing and spatial scale	*Indicate the start and stop dates of data collection, noting the frequency and periodicity of sampling and providing a rationale for these choices. If there is a gap between collection periods, state the dates for each sample cohort. Specify the spatial scale from which the data are taken*
Data exclusions	*If no data were excluded from the analyses, state so OR if data were excluded, describe the exclusions and the rationale behind them, indicating whether exclusion criteria were pre-established.*
Reproducibility	*Describe the measures taken to verify the reproducibility of experimental findings. For each experiment, note whether any attempts to repeat the experiment failed OR state that all attempts to repeat the experiment were successful.*
Randomization	*Describe how samples/organisms/participants were allocated into groups. If allocation was not random, describe how covariates were controlled. If this is not relevant to your study, explain why.*
Blinding	*Describe the extent of blinding used during data acquisition and analysis. If blinding was not possible, describe why OR explain why blinding was not relevant to your study.*

Did the study involve field work? ☐ Yes ☐ No

Field work, collection and transport

Field conditions	*Describe the study conditions for field work, providing relevant parameters (e.g. temperature, rainfall).*
Location	*State the location of the sampling or experiment, providing relevant parameters (e.g. latitude and longitude, elevation, water depth).*
Access and import/export	*Describe the efforts you have made to access habitats and to collect and import/export your samples in a responsible manner and in compliance with local, national and international laws, noting any permits that were obtained (give the name of the issuing authority, the date of issue, and any identifying information).*
Disturbance	*Describe any disturbance caused by the study and how it was minimized.*

Reporting for specific materials, systems and methods

We require information from authors about some types of materials, experimental systems and methods used in many studies. Here, indicate whether each material, system or method listed is relevant to your study. If you are not sure if a list item applies to your research, read the appropriate section before selecting a response.

Materials & experimental systems

n/a	Involved in the study	
☒	☐	Antibodies
☒	☐	Eukaryotic cell lines
☒	☐	Palaeontology
☒	☐	Animals and other organisms
☐	☒	Human research participants
☒	☐	Clinical data

Methods

n/a	Involved in the study	
☒	☐	ChIP-seq
☒	☐	Flow cytometry
☒	☐	MRI-based neuroimaging

Palaeontology

Specimen provenance	*Provide provenance information for specimens and describe permits that were obtained for the work (including the name of the issuing authority, the date of issue, and any identifying information).*
Specimen deposition	*Indicate where the specimens have been deposited to permit free access by other researchers.*
Dating methods	*If new dates are provided, describe how they were obtained (e.g. collection, storage, sample pretreatment and measurement), where they were obtained (i.e. lab name), the calibration program and the protocol for quality assurance OR state that no new dates are provided.*

☐ Tick this box to confirm that the raw and calibrated dates are available in the paper or in Supplementary Information.

Human research participants

Policy information about studies involving human research participants

Population characteristics

Human study 1: This cohort included 585 healthy lean (329 men and 256 women, 53.8±5.6 years old), 419 healthy overweight/obese (229 men and 190 women, 52.9±10.1 years old) and 103 overweight/obese diabetic (52 men and 51 women, 53.4±10.0 years old) participants.
Human study 2: A group of 91 subjects including 26 healthy controls (9 men and 17 women, 58.7±12.6 years old), 30 pre-diabetic individuals (10 men and 20 women, 61.6±13.1 years old) and 35 diabetic patients (16 men and 19 women, 63.0±8.5 years old) were recruited for this study.
Human study 3: A total of 38 obese diabetic patients (18 men and 20 women, 44.9±12.6 years old) who received Roux-en-Y gastric bypass surgery were enrolled.
Human study 4: Among all of the 132 subjects who were metabolically healthy at baseline (the year 2000-2001), 86 participants (26 men and 60 women, 32.8±9.6 years old) became metabolically unhealthy and 46 participants (10 men and 36 women, 33.2 ±6.2 years old) remained healthy.
Human study 5: Among all of the 207 subjects were metabolically healthy at baseline (the year 2011), 90 participants (60 men and 30 women, 39.5±10.4 years old) became metabolically unhealthy and 117 participants (57 men and 60 women, 40.0±10.4 years old) remained healthy.

Recruitment

Written informed consent was obtained from all participants before recruitment. Individuals with BMI < 25 kg/m2 were considered lean and those with BMI " 25 were classified as overweight/obese. Individuals with 6.1 mmol/L ! fasting blood glucose < 7.0 mmol/L or 7.8 mmol/L ! oral glucose tolerance test (OGTT) (2 h) < 11.1 mmol/L were classified as pre-diabetic. Subjects with fasting blood glucose " 7.0 mmol/L and/or OGTT (2 h) " 11.1 mmol/L were classified as diabetic. Subjects were considered “metabolically healthy” if they met all of the following criteria: fasting blood glucose < 6.1 mmol/L, OGTT (2 h) < 7.8 mmol/L and no previous history of diabetes; SBP/DBP <140/90 mmHg and no previous history of high blood pressure; fasting plasma TG < 1.7 mmol/L and fasting plasma HDL " 0.9 mmol/L (men) or " 1.0 mmol/L (women), and no previous history of high cholesterol (TC < 5.18 mmol/L); no history of cardiovascular or endocrine disease. Those who failed to meet all criteria above were classified as “metabolically unhealthy”.
Human study 1: It is a nested case-control study, performed within the Shanghai Obesity Study (SHOS) cohort, which was designed to investigate the occurrence and development of the metabolic syndrome and its related diseases. Beginning in 2009, the SHOS recruited participants from four communities in Shanghai, China. The participants that were selected satisfied the criteria for overweight/obese and diabetes. The exclusion criteria were: type 1 diabetes, pregnancy, severe diabetic complications (diabetic retinopathy, diabetic neuropathy, diabetic nephropathy and diabetic foot); severe hepatic diseases including chronic persistent hepatitis, liver cirrhosis or the co-occurrence of positive hepatitis and abnormal hepatic transaminase; severe organic disease, including cancer, coronary heart disease, renal disease, thyroid disease, myocardial infarction or cerebral apoplexy; infectious disease; alcoholism; and continuous medication (including weight loss or psychotropic medication) for over 3 d prior to enrollment.
Human study 2: The exclusion criteria were the same as in human study 1.
Human study 3: Any patient with a history of open abdominal surgery, a serious disease (such as heart or lung insufficiency) that was incompatible with surgery, an acute T2DM complication, severe alcohol or drug dependency, a mental disorder, type 1 diabetes, secondary diabetes, an unstable psychiatric illness, or who was at a relatively high surgical risk (such as a patient with an active ulcer) was excluded.
Human study 4: The subjects were selected from the Shanghai Diabetes Study, which was intended to assess the prevalence of diabetes and diabetes-associated metabolic disorders in urban Shanghai. They were metabolically healthy at baseline (the year 2000-2001). Ten years later (year 2010-2011), some participants became metabolically unhealthy (future metabolically unhealthy) and the others remained healthy (future metabolically healthy).
Human study 5: The subjects were collected from Institute of Basic Theory of Chinese Medicine, China Academy of Chinese Medical Sciences. These subjects were metabolically healthy at baseline (the year 2011). Five years later (the year 2016), some became metabolically unhealthy (future metabolically unhealthy) and the others remained healthy (future metabolically healthy).
The participants were selected satisfying the criteria for overweight/obese and diabetes and exclusion criteria. They were then grouped based on their clinical indices. The bias might be introduced for different age, sex, BMI or other indices among groups. To eliminate the confounding effects of age, sex, and BMI, we selected the samples with mathced age, sex and BMI.

Ethics oversight

The five human studies reported in this paper were all approved by the Ethics Committee of Shanghai Jiao Tong University Affiliated Sixth People's Hospital and Institute of Basic Theory of Chinese Medicine, China Academy of Chinese Medical Sciences in accordance with the World Medical Association’s Declaration of Helsinki.

Note that full information on the approval of the study protocol must also be provided in the manuscript.

ChIP-seq

Data deposition

☐ Confirm that both raw and final processed data have been deposited in a public database such as GEO.

☐ Confirm that you have deposited or provided access to graph files (e.g. BED files) for the called peaks.

Data access links
May remain private before publication.

For "Initial submission" or "Revised version" documents, provide reviewer access links. For your "Final submission" document, provide a link to the deposited data.

Files in database submission

Provide a list of all files available in the database submission.

Genome browser session
(e.g. UCSC)

Provide a link to an anonymized genome browser session for "Initial submission" and "Revised version" documents only, to enable peer review. Write "no longer applicable" for "Final submission" documents.

Methodology

Replicates

Describe the experimental replicates, specifying number, type and replicate agreement.

Sequencing depth

Describe the sequencing depth for each experiment, providing the total number of reads, uniquely mapped reads, length of reads and whether they were paired- or single-end.

Antibodies

Describe the antibodies used for the ChIP-seq experiments; as applicable, provide supplier name, catalog number, clone name, and lot number.

Peak calling parameters

Specify the command line program and parameters used for read mapping and peak calling, including the ChIP, control and index files used.

Data quality

Describe the methods used to ensure data quality in full detail, including how many peaks are at FDR 5% and above 5-fold enrichment.

Software

Describe the software used to collect and analyze the ChIP-seq data. For custom code that has been deposited into a community repository, provide accession details.

Flow Cytometry

Plots

Confirm that:

☐ The axis labels state the marker and fluorochrome used (e.g. CD4-FITC).

☐ The axis scales are clearly visible. Include numbers along axes only for bottom left plot of group (a 'group' is an analysis of identical markers).

☐ All plots are contour plots with outliers or pseudocolor plots.

☐ A numerical value for number of cells or percentage (with statistics) is provided.

Methodology

Sample preparation

Describe the sample preparation, detailing the biological source of the cells and any tissue processing steps used.

Instrument

Identify the instrument used for data collection, specifying make and model number.

Software

Describe the software used to collect and analyze the flow cytometry data. For custom code that has been deposited into a community repository, provide accession details.

Cell population abundance

Describe the abundance of the relevant cell populations within post-sort fractions, providing details on the purity of the samples and how it was determined.

Gating strategy

Describe the gating strategy used for all relevant experiments, specifying the preliminary FSC/SSC gates of the starting cell population, indicating where boundaries between "positive" and "negative" staining cell populations are defined.

☐ Tick this box to confirm that a figure exemplifying the gating strategy is provided in the Supplementary Information.

Magnetic resonance imaging

Experimental design

Design type

Indicate task or resting state; event-related or block design.

Design specifications

Specify the number of blocks, trials or experimental units per session and/or subject, and specify the length of each trial or block (if trials are blocked) and interval between trials.

Behavioral performance measures

State number and/or type of variables recorded (e.g. correct button press, response time) and what statistics were used to establish that the subjects were performing the task as expected (e.g. mean, range, and/or standard deviation across subjects).

Acquisition

Imaging type(s) — *Specify: functional, structural, diffusion, perfusion.*

Field strength — *Specify in Tesla*

Sequence & imaging parameters — *Specify the pulse sequence type (gradient echo, spin echo, etc.), imaging type (EPI, spiral, etc.), field of view, matrix size, slice thickness, orientation and TE/TR/flip angle.*

Area of acquisition — *State whether a whole brain scan was used OR define the area of acquisition, describing how the region was determined.*

Diffusion MRI ☐ Used ☐ Not used

Preprocessing

Preprocessing software — *Provide detail on software version and revision number and on specific parameters (model/functions, brain extraction, segmentation, smoothing kernel size, etc.).*

Normalization — *If data were normalized/standardized, describe the approach(es): specify linear or non-linear and define image types used for transformation OR indicate that data were not normalized and explain rationale for lack of normalization.*

Normalization template — *Describe the template used for normalization/transformation, specifying subject space or group standardized space (e.g. original Talairach, MNI305, ICBM152) OR indicate that the data were not normalized.*

Noise and artifact removal — *Describe your procedure(s) for artifact and structured noise removal, specifying motion parameters, tissue signals and physiological signals (heart rate, respiration).*

Volume censoring — *Define your software and/or method and criteria for volume censoring, and state the extent of such censoring.*

Statistical modeling & inference

Model type and settings — *Specify type (mass univariate, multivariate, RSA, predictive, etc.) and describe essential details of the model at the first and second levels (e.g. fixed, random or mixed effects; drift or auto-correlation).*

Effect(s) tested — *Define precise effect in terms of the task or stimulus conditions instead of psychological concepts and indicate whether ANOVA or factorial designs were used.*

Specify type of analysis: ☐ Whole brain ☐ ROI-based ☐ Both

Statistic type for inference
(See Eklund et al. 2016) — *Specify voxel-wise or cluster-wise and report all relevant parameters for cluster-wise methods.*

Correction — *Describe the type of correction and how it is obtained for multiple comparisons (e.g. FWE, FDR, permutation or Monte Carlo).*

Models & analysis

n/a	Involved in the study
☐	☐ Functional and/or effective connectivity
☐	☐ Graph analysis
☐	☐ Multivariate modeling or predictive analysis

Functional and/or effective connectivity — *Report the measures of dependence used and the model details (e.g. Pearson correlation, partial correlation, mutual information).*

Graph analysis — *Report the dependent variable and connectivity measure, specifying weighted graph or binarized graph, subject- or group-level, and the global and/or node summaries used (e.g. clustering coefficient, efficiency, etc.).*

Multivariate modeling and predictive analysis — *Specify independent variables, features extraction and dimension reduction, model, training and evaluation metrics.*
